# Assessing the Impacts of Recent Crop Expansion on Water Quality in the Missouri River Basin Using the Soil and Water Assessment Tool

**DOI:** 10.1029/2020ms002284

**Published:** 2021-05-28

**Authors:** Pan Chen, Yongping Yuan, Wenhong Li, Stephen D. LeDuc, Tyler J. Lark, Xuesong Zhang, Christopher Clark

**Affiliations:** 1 College of Water Resources Science and Engineering, Taiyuan University of Technology, Taiyuan, China; 2 Oak Ridge Institute for Science and Education (ORISE), USEPA, Durham, NC, USA; 3 Earth and Ocean Sciences, Nicholas School of the Environment, Duke University, Durham, NC, USA; 4 USEPA–Office of Research and Development, Durham, NC, USA; 5 Nelson Institute Center for Sustainability and the Global Environment, DOE Great Lakes Bioenergy Research Center, University of Wisconsin Madison, Madison, WI, USA; 6 Earth System Science Interdisciplinary Center, University of Maryland, College Park, MD, USA; 7 USEPA–Office of Research and Development, Washington, DC, USA

## Abstract

The Missouri River Basin (MORB) has experienced a resurgence of grassland conversion to crop production, which raised concerns on water quality. We applied the Soil and Water Assessment Tool (SWAT) to address how this conversion would impact water quality. We designed three crop production scenarios representing conversion of grassland to: (a) continuous corn; (b) corn/soybean rotation; and (c) corn/wheat rotation to assess the impact. The SWAT model results showed: (a) the lower MORB produced high total nitrogen (TN) and total phosphorus (TP) load before conversion (baseline) due mainly to high precipitation and high agricultural activity; (b) the greatest percentage increases of TN and TP occurred in the North and South Dakotas, coinciding with the highest amount of grassland conversion to cropland; and (c) grassland conversion to continuous corn resulted in the greatest increase in TN and TP loads, followed by conversion to corn/soybean and then conversion to corn/wheat. Although the greatest percentage increases of TN and TP occurred in the North and South Dakotas, these areas still contributed relatively low TN and TP to total basin loads after conversion. However, watersheds, predominantly in the lower MORB continued to be “hotspots” that contributed the greatest amounts of TN and TP to the total basin loads—driven by a combination of grassland conversion, high precipitation, and loading from pre-existing cropland. At the watershed outlet, the TN and TP loads were increased by 6.4% (13,800 t/yr) and 8.7% (3,400 t/yr), respectively, during the 2008–2016 period for the conversion to continuous corn scenario.

## Plain Language Summary

This is a large-scale environmental assessment of how land use/land cover changes impact water quality. The US Midwest has undergone a cropland increase of corn and soybean production from 2008 to 2016. Most of this cropland increase has displaced grasslands, which has raised concerns on water quality and ecosystems. In this study, a large-scale watershed model, the Soil and Water Assessment Tool (SWAT), was applied to the Missouri River Basin (MORB), where some of the highest rates of grassland conversion to cropland have occurred. The SWAT model was used to quantify water quality changes resulting from grassland conversion to cropland. Results from this study showed that conversion from grassland to cropland increased nutrient loading to waterbodies. In addition, this study identified MORB areas within states such as Iowa, Missouri, Nebraska, and Kansas as producing the greatest amounts of nutrient loading due to a combination of grassland conversion, high precipitation, and high percentage of pre-existing cropland.

## 1. Introduction

The US Midwest has undergone a profound shift in land use and land cover (LULC) in recent decades. After a 25-year decrease from 1982 to 2007, total cropland increased by a net of 3.9 million acres between 2007 and 2012 (US Department of Agriculture [USDA], 2015). Most of this increase came from grasslands, including pasture and hay (Arora & Wolter, 2018; Spawn et al., 2019; USDA, 2015). Lark et al. (2015) estimated a net increase of 3 million acres of total cropland between 2008 and 2012 nationally. Most of these newly converted lands (77%) were former grasslands, and the first crop planted (or “breakout crop”) consisted primarily of corn (27%), wheat (25%), or soybeans (20%) (Lark et al., 2015). This increase in cropland is still relatively small compared to the total US agricultural land base yet, at its maximum, roughly equals the size of New Jersey. Moreover, much of this increase has occurred on marginal lands, which generally are more erodible and less productive as defined by the USDA’s Natural Resources Conservation Service (NRCS), with potentially larger environmental impacts relative to prime agricultural fields (Lark et al., 2015, 2020).

Some of these LULC changes may be associated with biofuel feedstock production of corn and soybeans. For example, ethanol production increased from 2.1 to 14.3 billion gallons during 2002–2014, and almost all gasoline sold in the US contains ethanol, with more than 90% of it produced from domestically grown corn grain (Hoekman et al., 2018). Similarly, biodiesel production from soybeans increased nearly 160-fold from 2001 to 2013 (Energy Information Administration, 2013). Furthermore, among these LULC changes, higher rates of land conversion were found around biorefineries (Wright et al., 2017), which suggested a potential contributing role of biofuels in the LULC trends observed. In addition to biofuels, other socio-economic factors such as population pressure, historically high corn prices in the 2007–2008 time period (USDA, 2015) and policy change also likely affected the LULC dynamics.

Studies focused on the environmental implications of increasing biofuel production have shown that LULC changes, particularly grassland conversion to crops, would increase nutrient loadings to surface waters (Farrell et al., 2006; Taguas et al., 2017; Wu & Zhang, 2015; Zhang et al., 2021), which have been a great concern worldwide. Water bodies and coastal areas around the world are threatened by excessive amounts of nutrients from upstream watersheds, which can cause rapid proliferation of algae as seen in areas of Lake Erie and the Northern Gulf of Mexico (Alexander et al., 2008; Jones et al., 2018; NSTC, 2000; Rabalais et al., 2001; USEPA, 2014; Yuan et al., 2018). These algal blooms negatively impact drinking water sources, aquatic species, and recreational services of water bodies by producing toxins, also called harmful algal blooms. Thus, quantifying how the increased crop production would impact nutrient loadings and finding ways of reducing nutrient losses from agricultural fields are of paramount importance.

A number of studies have attempted to evaluate the changes of water quantity and quality in response to different future scenarios of LULC driven by biofuel development (Deb et al., 2015; Gu et al., 2015; Panagopoulos et al., 2014, 2017; Wu & Zhang, 2015; Yu & McCarl, 2018). These research studies assessed the potential environmental impacts of possible biofuel scenarios and provided useful information for selecting environmentally friendly bioenergy crops. For example, Panagopoulos et al. (2014) evaluated the potential effects of four different future scenarios on water quality for the Upper Mississippi River Basin (UMRB). The four scenarios evaluated were: (a) expansion of continuous corn across the entire basin; (b) adoption of no-till practices on corn/soybean production; (c) conversion of corn/soybean rotation to corn, soybean, and three years of alfalfa; and (d) implementation of winter cover cropping. Their results indicated that continuous corn would result in increased N loss to water bodies while other measures were environmentally effective for reducing sediment, N, and P losses. Deb et al. (2015) showed that converting cropland to switchgrass could reduce erosion and N loading. Yu and McCarl (2018) analyzed the effects of land use change on water quantity and quality based on a land use model, the Forest and Agricultural Sector Optimization Model with Greenhouse Gases (FASOM-GHG), and showed that increases in crop land use significantly degrade water quality. Wu and Zhang (2015) also found that increased crop land would degrade water quality, but increasing the amount of switchgrass acreage would mitigate the nutrient loads. Those studies based on future alternative scenarios were helpful in understanding potential environmental impacts of LULC changes. However, the environmental impacts, particularly water quality impacts, that have already resulted from past/ongoing LULC changes have not yet been studied. Therefore, the goal of this study was to evaluate water quality impacts due to cropland expansion driven by biofuel and commodity market that have already taken place. These estimates will be critical in determining future directions in targeting biofuel production schemes.

Monitoring programs are often used to evaluate land management effects on nonpoint source pollution (Shih et al., 1994). Long-term monitoring better reflects multi-year climatic variability and helps assure that a range of events and conditions are covered (Borah & Bera, 2003; Stone et al., 2000). Because long-term monitoring is expensive and often limited by personnel and financial resources, short-term monitoring with complimentary simulation modeling may be used as an alternative for environmental assessment.

Models such as the USDA-Agricultural Research Service Soil and Water Assessment Tool (SWAT) (Gassman et al., 2007; Neitsch et al., 2011) have been developed to aid in the evaluation of watershed response to agricultural management practices. The model has been widely applied to evaluate best management practices, alternative land use/land management, and climate change on runoff and pollutant losses to streams within a watershed (Baffaut et al., 2015; Chaplot et al., 2004; Du et al., 2018; Gassman et al., 2007; Johnson et al., 2015; Niraula et al., 2013; Rajib & Merwade, 2017; Santhi et al., 2006; Vaché et al., 2002). The model has also been used to explore the relationships between potential increases in biofuel production, land conversion, and impacts on water quality (Gassman et al., 2007; Panagopoulos et al., 2017; Wu & Zhang, 2015). Thus, the SWAT model was selected for this study. The detailed objectives of this study were to: (a) quantify nutrient loading changes for the entire Missouri River Basin (MORB); (b) estimate changes in water quality metrics per unit area of land use; and (c) identify hot spots experiencing the greatest increase in nutrient loadings. Results from this study will help better plan future biofuel targets. In addition, government incentives may be needed to reach the targets without further degrading water quality.

## 2. Methods and Procedures

### 2.1. Study Area

**Figure 1.**
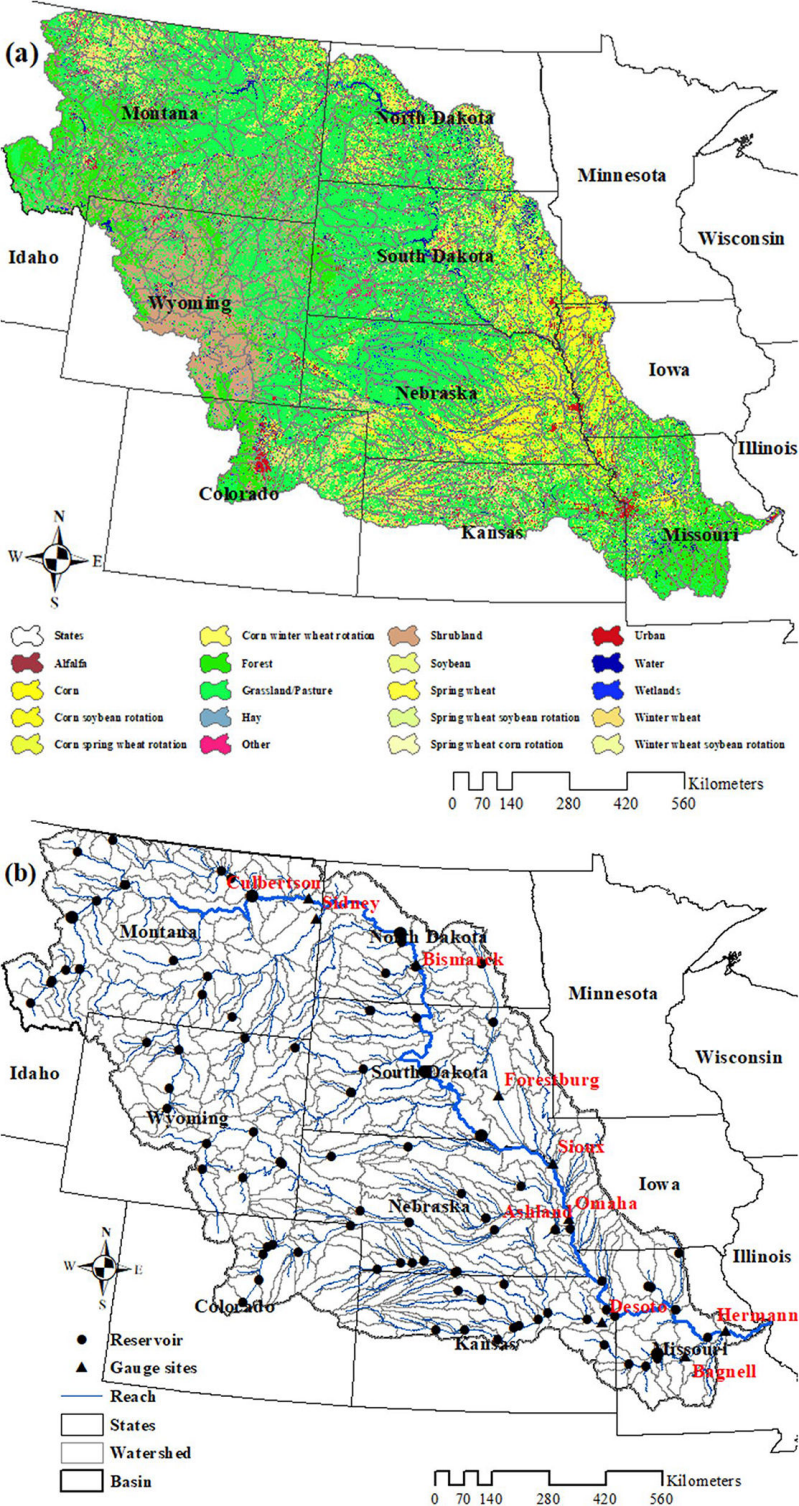
(a) Land use/Land cover, and (b) reaches, watersheds, and available US Geological Survey gauge sites of the Missouri River Basin (MORB).

The MORB was selected to estimate the water quality changes resulting from the recent shift in land use from grassland to crops because of the following reasons: (a) the MORB is one of the largest sources of nutrients to the Gulf of Mexico due to increased fertilizer runoff (Demissie et al., 2012; Wu et al., 2012); and (b) some of the highest rates of grassland conversion have occurred within this watershed, particularly along the western edge of the Corn Belt in the eastern Dakotas (Lark et al., 2015). The watershed covers approximately 1.3 million km^2^ and includes 10 US states and part of Canada (which contains about 2% of the MORB’s total area). The Missouri River originates from the Rocky Mountains of west Montana (MT) and confluences with the Mississippi River near St. Louis with a main stem length of nearly 3,800 km. The largest tributaries of the MORB include the Yellowstone, Platte, and KS Rivers; all have a drainage area greater than 150,000 km^2^ and average annual runoff greater than 190 m^3^/s (Wu & Zhang, 2015). Rangeland located in the western and central MORB is the dominant land cover, accounting for about half of the total watershed area (Yu & McCarl, 2018). Cropland concentrates in the eastern and southern parts of the basin and accounts for a quarter of the total area. Major crop types include corn, soybean, winter wheat, and spring wheat (Figure 1a). The rest of the watershed area consists of shrubland (10%), forest (9%), urban areas (3%), wetland (1%), water (1%) and barren land (<1%).

The MORB is extremely diverse in many respects as a large basin. The Rocky Mountains, which form the western boundary, have an exceptionally rugged topography. Its geography varies from the mountains of Colorado (CO), MT, and Wyoming (WY), with the elevation of some peaks more than 4,399 m above sea level, to the low lands of Missouri (MO) with an elevation of less than 120 m (Qiao et al., 2014). Climate varies from arid and semi-arid to sub-humid. Most of the basin receives an annual average of 200–250 mm of precipitation. However, the westernmost parts of the basin in the Rockies and the southeastern portions in MO may receive as much as 1,000 mm per year. There is also a wide range of temperatures in the region. The temperature in MT can drop to −51°C in winter, while it can reach to 49°C in MO in summer (Zhang & Wu, 2013). Soil types within the basin include well drained, moderately well drained, and poorly drained soils, from northwest to southeast (Flynn et al., 2017). The geographic extent of the basin includes parts of MT, WY, CO, North Dakota (ND), South Dakota (SD), Nebraska (NE), Kansas (KS), Iowa (IA), and MO (Figure 1b).

### 2.2. SWAT Model Description

The SWAT (Arnold et al., 1998; Neitsch et al., 2011) was applied in the MORB to assess impacts of the recent land use shift from grassland to crops on hydrology and water quality. The SWAT model is a continuous, long-term, physically based distributed model developed to assess the impacts of climate and land use and management changes on hydrology, sediment, and nutrients processes in watersheds (Arnold et al., 1998; Neitsch et al., 2011). In the model, a watershed or basin is divided into subwatersheds or subbasins. Subbasins are further divided into a series of uniform hydrological response units (HRUs) based on LULC, soil type, and slope. Hydrological components, sediment and nutrient yields are simulated for each HRU and then aggregated for the subbasins (Gassman et al., 2007; Neitsch et al., 2011; Williams et al., 2008).

The detailed SWAT simulations of hydrological components, sediment and nutrient yields can be found in the SWAT theoretical manual (Neitsch et al., 2011). Briefly, hydrological components simulated in the model include evapotranspiration, surface runoff (SURQ), percolation, lateral flow, groundwater flow (return flow), transmission losses, and ponds (Arnold et al., 1998). The SURQ is estimated using a modification of the SCS (Soil Conservation Service, now the Natural Resources Conservation Resource) curve number method (Arnold et al., 1998) with daily rainfall amounts. The curve number values are based on soil type, LULC, and land management conditions (Rallison & Miller, 1981) and are adjusted according to soil moisture conditions (Arnold et al., 1998). Sediment yield is calculated with the Modified Universal Soil Loss Equation method (Williams & Berndt, 1977). For nutrients, SWAT simulates two broad categories of nutrients: organic and dissolved forms of N and P. The former includes active, stable, and fresh organic N and P, and the latter contains ammonium (NH_4_^+^), nitrate (NO_3_^−^), nitrite (NO_2_^−^), mineral and soluble P (Neitsch et al., 2011). Nutrients are mainly added to the soil by plant residue and fertilizer, and are removed by plant uptake, and runoff and sediment loss. The crop residues are left on the ground after being harvested, and then converted to organic nutrients and added to the soil through decomposition and mineralization processes. The organic and dissolved nutrients can be directly added in the soil by fertilizer application (Neitsch et al., 2011). After rainfall, runoff may be generated, and it carries dissolved N and P as it flows off the fields. In addition, organic nutrients (N and P) and mineral P attached to the soil may be transported with sediment off the fields.

The SWAT model also has channel components. More details on channel components can be found in the SWAT theoretical manual (Neitsch et al., 2011). Briefly, the water movement at the channels is routed with the storage routing variable. Once the sediment yield is estimated, sediment transport in the channel network is simulated with the simplified version of Bagnold’s equation, and both deposition and degradation are simulated (Bagnold, 1977). The nutrient movement in the channel is modeled with a water quality model, QUAL2E, and growth and decay of algae, settling of organic N and P, and water temperature are considered in the simulation (Brown & Barnwell, 1987).

### 2.3. Data Collection

The input data of the SWAT model included elevation, soil, land use, land management, and weather. In addition, monitored streamflow and water quality data were also needed for model calibration and validation. Most of the data used for this study were publicly available and are described in Table 1 and further below.

#### Elevation and Soil:

The elevation data were represented by a 90-m US Geological Survey (USGS) Digital Elevation Model (DEM). The 90-m DEM resolution was used to reduce computation time due to the size of the basin studied. The State Soil Geographic (STATSGO) Database was used to define the properties and distribution of soils. There were 1,804 different soil types within the MORB based on STATSGO. The soil physical properties used in SWAT included texture, bulk density, available water capacity, saturated hydraulic conductivity, and soil albedo.

#### Land use and land management.

The USDA National Agricultural Statistics Service (NASS) 2008 and 2009 Cropland Data Layers (CDL) were acquired and used to estimate LULC (USDA-NASS, 2016). The CDL was selected because of the fine level of detail it provided for specific crop and land cover classes and their rotations. The classes of interest for this study were row crops, which included corn, soybeans, and wheat, as well as grassland/pasture. Detailed information of agricultural land management was obtained from USDA (https://nassgeodata.gmu.edu/CropScape/).

#### Weather data:

Historic daily precipitation and maximum and minimum temperatures of 1,721 National Weather Service (NWS) stations across the study region were obtained from the National Oceanic and Atmospheric Administration and National Climatic Data Center (NCDC, 2016). The weather data were then screened for missing values, and interpolated from the nearest weather station with available data. Other weather data, like wind speed, relative humidity, and solar radiation data, were generated internally by the SWAT model.

#### Point source:

Loads of mineral N and P released to the rivers of MORB from thousands of point sources across the region were obtained from the Hydrologic and Water Quality System database (Schwarz et al., 2006; Yen et al., 2016) and added to the appropriate subwatersheds. These point sources were simulated with the constant daily load method at each 8-digit subbasin.

#### Fertilizer input:

The Input Editor of the Annualized Agricultural Nonpoint Source Pollutant Model was used to generate the crop management files for the SWAT model based on the template files in the Revised Universal Soil Loss Equation 2 (RUSLE2) developed by the USDA NRCS (Chiang et al., 2014). Most of the operations, including planting, tillage, and harvesting, were recorded in these crop management zone template files, except for the fertilizer application. Fertilizer application rates for corn, soybean, and wheat were calculated based on data from the Census of Agriculture of USDA-NASS (Table 2).

#### Tillage.

Estimates of the distributions of four tillage types, including no-till, mulch, reduced, and intense, were obtained at the 8-digit watershed level and added in the SWAT model (Baker, 2011). We selected the most common data on tillage (Baker, 2011) to best align with previous studies. The mixing efficiency of 5%, 35%, 65% and 95% and tillage depth of 25, 50, 100, and 150 cm were set for each tillage type (low value for no-till and high value for intense tillage).

#### Reservoir.

There are many reservoirs in the MORB. Information on reservoirs including surface area of the reservoir and normal and maximum storage of the reservoir were obtained from the national inventory of dams (US Army Corps of Engineers USACE, 2012). Downstream discharge was monitored for a few reservoirs on the main stream of MORB, and it was used as an input to the model for daily release. For the rest reservoirs, no detailed operation information such as release rate and timing were available, thus a monthly target release method was applied. From the thousands of reservoirs included in the inventory data, the first 100 largest reservoirs were included for the MORB models, because SWAT limited the number of reservoirs that could be included. However, this number was sufficient to reproduce the potential reservoir impacts on water flow and pollutant movement in the rivers, as the reservoirs chosen were the largest and occupied the vast majority (around 94%) of total artificial water volume in the study area (Daggupati et al., 2016).

#### Streamflow and water quality.

The USGS streamflow gauging stations (Figure 1b) were screened for observed data availability, length, and coverage of the data record. The final number of calibration sites for the MORB was 10 (Table 3). Monthly streamflow and available water quality data from 1975–2016 were downloaded from the 10 USGS gauging stations.

### 2.4. Model Parameterization

One of the key aspects of the SWAT model parameterization is the delineation of the study basin into many subbasins, which are further divided into a series of HRUs to depict the wide range of slopes, soils, land use, and land management that exists in the basin. Usually, multiple HRUs would be defined within a subbasin based on the thresholds for slope, soil, and land use. However, the “Dominant HRU” method is often suggested to reduce the requirement for computational power in large-scale SWAT modeling (Daggupati et al., 2016; Panagopoulos et al., 2015). Both Daggupati et al. (2016) and Panagopoulos et al. (2015) adopted this method in their UMRB and MORB studies, respectively, and found that using 12-digit hydrologic units (HUC-12) boundaries as subbasins with dominant HRU for each HUC-12 captured important climate and topographical variabilities within the basin and produced satisfactory hydrologic and water quality estimates, although land use variation was not accurately represented.

Since the objective of this study was to evaluate water quality impacts of land use change across the MORB, attempts were made to better capture land use changes from 2008 to 2016. Therefore, we used multiple HRUs per basin to capture land use variation, and set a 0% minimum threshold for land use in order to capture all changes. To balance the need for capturing the detailed land use changes and computational requirement, 8-digit hydrologic units (HUC8s) defined by the USGS (http://water.usgs.gov/GIS/huc.html) were selected as SWAT subbasins. In total, the MORB included 304 subbasins (HUC8s). The stream network of the NHDPlus data-set developed by the USGS and USEPA (http://epa.gov/waters) was used to determine preferred flow paths between the subbasins. Each of the subbasins was further divided into several spatially uniform HRUs based on land use, soil type, and slope. Thresholds of 0%, 10%, and 5% were used for land use, soil, and slope, respectively, resulting in a total of 56,424 HRUs in the MORB.

As the goal of this research was to assess the effects of land use change on water quality, significant care was taken to define land use/land management categories. The Representative Crop Rotations Using Edit Distance algorithm was used to select representative crop rotations by combining and analyzing the CDLs (Sahajpal et al., 2014). First, CDLs from 2008 to 2009 were combined and 1,201 different rotations were obtained for the MORB. Second, the area for each rotation and its percentage were calculated and ranked in descending order. Third, the accumulative percentage of rotations from top to bottom was calculated until 90% of all cropland area was accounted for, resulting in 46 representative rotations. Rotations including corn, soybean, winter wheat, and spring wheat accounted for 90% of those 46 rotations, and thus we further reduced the 46 rotations to 23 types by combining the small percentage of remaining rotations with those they were most similar to. For example, durum wheat was assumed as spring wheat, so spring and durum wheat were combined into the single management category spring wheat. Likewise, as peas are managed similarly to soybeans, the two were combined into the soybean management category. The final land use rotation information is shown within the supporting information of Table S1.

### 2.5. Model Calibration

SWAT-CUP offers several algorithms for parameter calibration (Abbaspour et al., 2015). The Sequential Uncertainty Fitting (SUFI-2) algorithm, used in other large-scale studies (Pagliero et al., 2014; Panagopoulos et al., 2015), was selected in this study for model calibration.

Two steps were taken in calibrating streamflow. In the first, a SWAT model with the same inputs but a coarser DEM (300 m) was set up. We manually adjusted the five snow parameters: snowfall temperature, snow melt base temperature, maximum and minimum melt rate for snow, and the snow pack temperature lag factor (Table S2 in the supporting information) first by checking the magnitude and shapes of snowmelt process in SWAT runs. Then, the remaining 11 parameters (Table S2), including four surface flow parameters (curve number 2 (CN2), soil evaporation compensation factor (ESCO), plant uptake compensation factor (EPCO), and available water capacity of the first soil layer (SOL_AWC(1))), five groundwater parameters (GW_DELAY, ALPHA_BF, GWQMN, GW_REVAP, and RCHRG_DP), and two channel parameters (CH_K2 and ALPHA_BNK), were auto-calibrated using the SWAT-CUP interface with SUFI-2 within 500 simulations. The CN2 and SOL_AWC(1) parameters were allowed to change by a percentage from their default values (± 20%), while others were modified with absolute values within realistic ranges (Panagopoulos et al., 2015). Those parameters were suggested by developers (Neitsch et al., 2011) and were commonly calibrated in published large-scale SWAT applications (Arnold et al., 1998; Van Liew & Garbrecht, 2003; White & Chaubey, 2005). During this process, we calibrated the subbasins, which were hydrologically independent (Table 3) simultaneously, and then adjusted the remaining four subbasins, which would receive streamflow and nutrients from upstream subbasins. In the second step, calibrated parameters were directly transferred to the finer resolution model with a 90-m DEM. The parameters were then manually fine-tuned to get more satisfying results.

Limited time-series of water quality data were available compared to streamflow data. For example, among those USGS gauging stations with streamflow monitoring, only three sites had water quality data. We calibrated the parameters related to sediment and nutrient manually and adjusted them at the basin scale. In the SWAT model, upland erosion and channel erosion are two main processes producing sediment yield. The upland erosion process was often adjusted by changing the USLE parameters, including the USLE soil erodibility factor (USLE_K), the USLE cover and management factor (USLE_C), and the USLE support practice factor (USLE_P), as in previous studies (Betrie et al., 2011; Mukundan et al., 2010). In this study, we adjusted the USLE_P value to 0.65, and used default values of USLE_K and USLE_C, which were determined by soil and crop types. The linear and exponential coefficients, the channel erodibility factor nd the channel cover factor, which control sediment deposition and degradation processes in the channel (Schilling et al., 2011), were changed to 1.5, 0.0012, 0.3, and 0.5, respectively. Organic N and nitrate-N are two important components of total nitrogen (TN). Accordingly, two parameters—including the organic N enrichment ratio, which is defined as the ratio of concentration of organic N transported with sediment to the concentration in the soil surface layer (Yuan and Chiang, 2015), and N percolation coefficient, which governs the amount of nitrate moved with runoff (Neitsch et al., 2011)—were adjusted to 2.2 and 0.7, respectively, in the calibration of TN loads. Similarly, the parameters for the organic P enrichment ratio and the P percolation coefficient, which control the amount of organic and soluble P (White et al., 2014), were changed to 1.6 and 15, respectively, to match the total phosphorus (TP) loads.

To accelerate the auto-calibration process with the use of SWAT-CUP and SUFI-2, the most recent 20-years (1996–2016) period was used for calibration, because the model was set up with 2008/2009 CDL land use data. Comparative data from 1975 to 1996 were used for validation. Streamflow and water quality predictions made by SWAT were compared with observed streamflow and water quality data at USGS gauges to evaluate the model’s hydrologic predictions. The monthly time-series of nutrients were calculated using the Load Estimator (Runkel et al., 2004). During model calibration and validation, observed and modeled values on a monthly basis were compared visually and quantitatively. For the quantitative approach, the coefficient of determination (*R*^2^), Nashe-Sutcliffe efficiency (NSE), and Percent bias (PBIAS) were used as evaluators of model performance:

(1) $$R^{2}=\frac{{\left(\sum_{i}\left(OV_{i}-\bar{OV}\right)\left(MV_{i}-\bar{MV}\right)\right)}^{2}}{\sum_{i}{\left(OV_{i}-\bar{OV}\right)}^{2}\sum_{i}{\left(MV_{i}-\bar{MV}\right)}^{2}}$$

(2) $$NSE=1.0-\frac{\sum_{i}{\left(OV_{i}-MV_{i}\right)}^{2}}{\sum_{i}{\left(OV_{i}-\bar{OV}\right)}^{2}}$$

(3) $$PBIAS=\frac{\sum_{i}\left(OV_{i}-MV_{i}\right)}{\sum_{i}OV_{i}}\times 100$$

Where OV_i_ and MV_i_ are the observed and simulated values at time step *i*, respectively; and $\bar{OV}$ and $\bar{MV}$ are the average observed and simulated values over the simulation period.

*R*^2^, NSE, and PBIAS are often used to evaluate model performance, but there are no rules to determine if a 0.5 value for any statistic is good or bad. Moriasi et al. (2015) indicate that the monthly fits between simulated and observed stream flows can be regarded as “successful” when the NSE and PBIAS for these individual fits are greater than 0.5 (>0.5) and less than 25 percent (<25%), respectively. Streamflow performance was then evaluated by determining the *R*^2^ and PBIAS for each streamflow guage station in this study. For sediment and nutrients, model simulations can be judged as satisfactory on a monthly scale, if PBIAS is measured up to ± 55% for sediment and ± 70% for N and P (Moriasi et al., 2015). However, more strict limits, ± 30% bias for sediment and ± 40% bias for nutrients, following with Santhi et al. (2014), were selected to obtain a more realistic simulation. The NSE and *R*^2^ of sediments and nutrients were also calculated, although they were not regarded as the critical indices for calibration success (Panagopoulos et al., 2015).

### 2.6. Land Use Scenarios of Cropland Expansion

**Figure 2.**
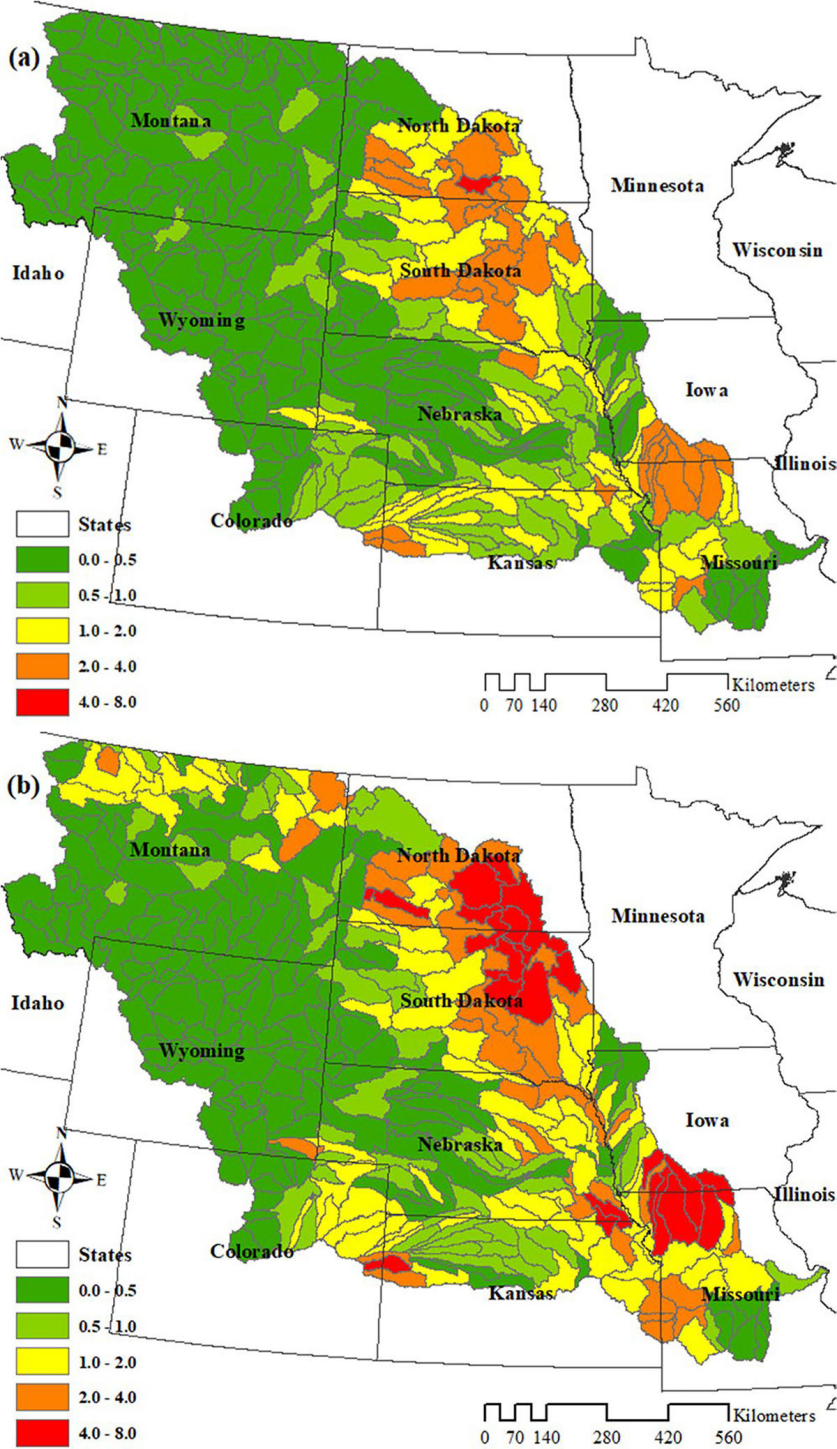
Percentage of area converted from noncrop land to crop land in each 8-digit hydrologic unit during (a) 2008–2012 and (b) 2008–2016.

Using the USDA CDLs from 2008 to 2012, Lark et al. (2015) tracked crop expansion pathways in the US. In their study, all CDL land use categories were first combined into two broad categories: crop and noncrop. The former class included corn, soybeans, wheat, cotton, etc., and the latter category encompassed forest, shrubland, wetland, and other noncrop lands. The five-year combinations of crop or noncrop were then applied to identify five different categories of land use over that interval: (a) Stable noncropland; (b) Stable cropland; (c) Conversion to cropland; (d) Conversion to noncropland; and (e) Intermittent cropland/uncertain. See Lark et al. (2015) for details of the method to identify these five categories and Lark et al. (2017, 2021) for information on their accuracy. Within the third category, conversion to cropland, they further identified the types and locations of crops planted on converted land spanning from 2008 to 2012. They found that corn was the most common crop planted on new land, including corn rotation with other crops such as soybean and wheat. In the US Corn Belt, SD and ND experienced the greatest amount of new cultivation in the MORB. There was wide variation of land use/land cover change rates in the entire MORB basin, ranging from none to 4.1% (HUC 10130104 in ND) (Figure 2a). More recently, Lark et al. extended their land use/land cover conversion data through the 2016 growing season and found that a similar pattern with additional changes occurred during the 2008–2016 period (Lark et al., 2020). The greatest change in percentage, 7.2%, occurred in the subbasin 10160004 in ND and SD from 2008 to 2016. On average, 0.77% of the total land area (1,318,712 km^2^) was converted from noncrop land to crop land during 2008–2012 (Figure 2a), and 1.18% of the total land area was converted from 2008 to 2016 (Figure 2b).

To evaluate the influence of land use/land cover change on water quality, four different simulation scenarios, including baseline, were designed. To construct the scenarios, we combined the 2008 and 2009 CDLs to capture at least two years of baseline crop rotation. Next, we overlaid this baseline data with locations of cropland conversion (Lark et al., 2015, 2020) to distinguish the areas where noncropland was converted to cropland. For simplification, we assumed grassland was the starting land cover for all converted locations, because 97% of those areas are Grass/Pasture and Other Hay/Non Alfalfa in our baseline map. Although the approximate locations of changes from noncrop land to crop land are known (Figures 2a and 2b), the exact rotations were not tracked. Therefore, we simulated three different post-conversion land use scenarios that represent the dominant crop rotations in the region: continuous corn, corn/soybean rotation, or corn/wheat rotation. Collectively, these three rotations account for 70% of the spatial and temporal crop patterns in the MORB and thus provide a realistic representation of the potential fate of converted land. All designed scenarios are listed in Table 4.

## 3. Results and Discussion

### 3.1. Model Calibration and Validation

**Figure 3.**
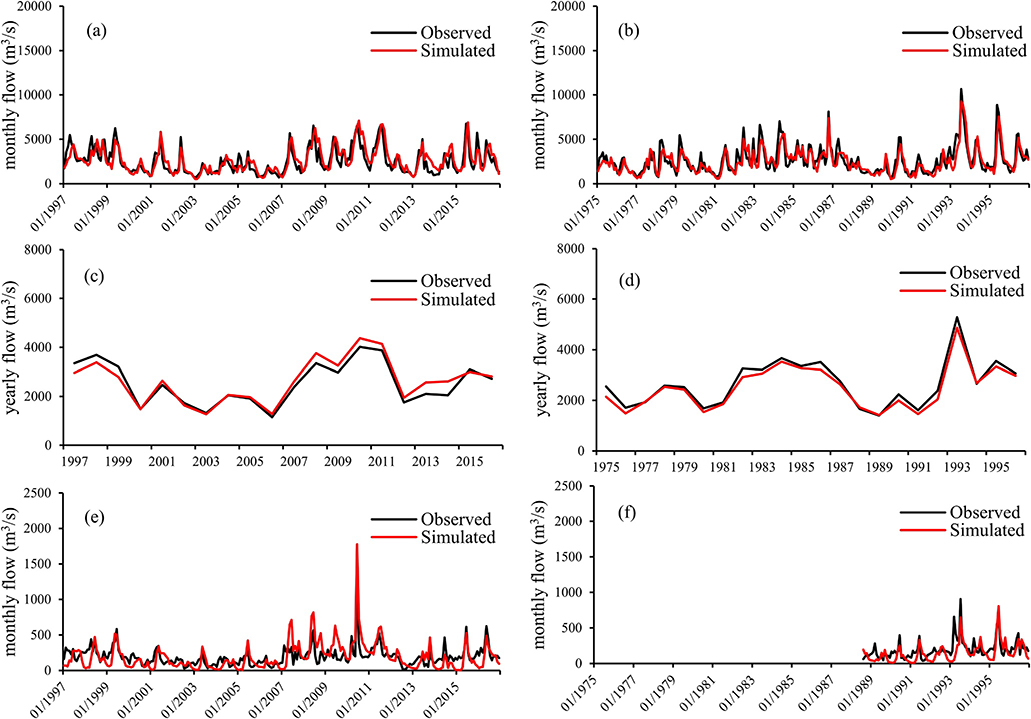
Monthly and yearly streamflow comparison between Soil and Water Assessment Tool (SWAT) simulated and monitored at the Hermann station (a and c) during calibration period 1997–2016 and (b and d) validation period 1975–1996, and (e and f) monthly streamflow comparison between SWAT simulated and monitored at the Ashland station during these two periods.

Model calibration and validation statistics of observed and simulated streamflow for all gauge stations are presented in Table 5 (at monthly scale) and 6 (at annual scale). *R*^2^ and NSE values were greater than 0.5 for all the USGS gauges except Ashland during the calibration period. In addition, most of the PBIAS values (Tables 5 and 6) indicated satisfactory to very good model performance. Time series comparison also demonstrated good agreement between simulated and observed streamflow across the MORB (Figures S1 and S2 in the supporting information). Particularly, most of the peaks and recession limbs in the hydrographs were well reflected in the SWAT simulations. The model performed very well at the MORB outlet, Hermann station (USGS station 069345004) for both monthly (Figures 3a and 3b) and annual streamflow (Figures 3c and 3d). The SWAT-simulated monthly streamflow followed seasonal trends of the observed streamflow, with an *R*^2^ of 0.7 and NSE of 0.67 for calibration and an *R*^2^ of 0.75 and NSE of 0.74 for validation (Table 5). Statistical calibration and validation results were even better for annual streamflow, with an *R*^2^ of 0.91 and NSE of 0.89 for calibration and an *R*^2^ of 0.98 and NSE of 0.95 for validation (Table 6).

Comparisons of simulated and observed monthly streamflow at the Ashland gauge station (USGS station 06801000) for the calibration period from 1997 to 2016 showed that SWAT-simulated streamflow generally followed seasonal trends of the observed streamflow (Figures 3e and 3f), although with an *R*^2^ of 0.51 and NSE of −0.11 (Table 5). The low NSE value of −0.11 indicated that the observed and predicted data did not fit the 1:1 line well. The SWAT model over-predicted peaks, particularly for high peaks, such as that of June 2010, which resulted in an over-prediction overall during the calibration period. The upstream area of the Ashland gauge station is located on the high groundwater recharge region of the Ogallala aquifer as documented in many other studies (Daggupati et al., 2016). The poor performance may be due to the extensive irrigation and frequent groundwater withdrawals in this region. SWAT over-estimation of streamflow for groundwater recharge dominant basins has also been reported in other studies (Wu & Johnston, 2008; Yuan et al., 2018).

The final values of main hydrologic parameters used in the MORB simulation model are summarized in Table S2 in the supporting information. One of the most critical parameters affecting streamflow generation is the CN. CN was increased for many of the upstream stations, such as Culbertson and Bismarck, while it was decreased for many of the downstream stations, such as Desoto and Bagnell, which indicated that the streamflow would be under-estimated using default CN values for the upstream MORB but over-estimated using default CNs for the downstream MORB. The ESCO values of Culbertson and Sidney were much larger than that of other stations, indicating that less water was extracted due to the evaporative demand of soil (Neitsch et al., 2011). EPCO values of stations in upstream areas of the MORB were also larger than those in downstream subbasins, a function of greater water uptake from the lower soil layers (Neitsch et al., 2011). Since less precipitation (200–250 mm) occurred in the upstream area of the MORB compared with other areas, surface soil in this region was very dry and thus less water was available for evaporation. To meet plant uptake demand, more water must be taken from the lower soil layers. Changes in SOL_AWC(1) did not present a pattern among different subbasins, similar to results of Panagopoulos et al.’s study in the Upper Mississippi River Basin and Ohio-Tennessee River Basin (2015). This may be due to the coarse resolution of the STATSGO soil database (Panagopoulos et al., 2015). Although effects of the five groundwater parameters on runoff were much smaller than those four SURQ parameters, adjustment of those groundwater parameters further improved SWAT simulations. The two channel parameters, CH_K2 and ALPHA_BNK, varied across the MORB (Table S2 in the supporting information), which was related to the extremely diverse topography in this relatively large basin.

Less data were available for model calibration and validation on sediment and nutrients. Among those 10 selected USGS gauging stations, Sidney, Sioux, and Omaha had sediment data for model evaluation and Sidney, Desoto, and Hermann had nutrient data for model evaluation. Since sediment movement is an important means of nutrient transportation, the SWAT model’s performance on sediment was evaluated first. SWAT model performance on sediment varied among the three stations; the model performed the best for Sidney but not as well for the other two stations (Tables 7 and 8). Similarly, SWAT model performance on nutrients also varied among stations. It performed the best for Hermann, the outlet of the MORB (Tables 7 and 8). However, the SWAT model performed the worst on TN and TP for Sidney, which had the best performance on sediment.

**Figure 4.**
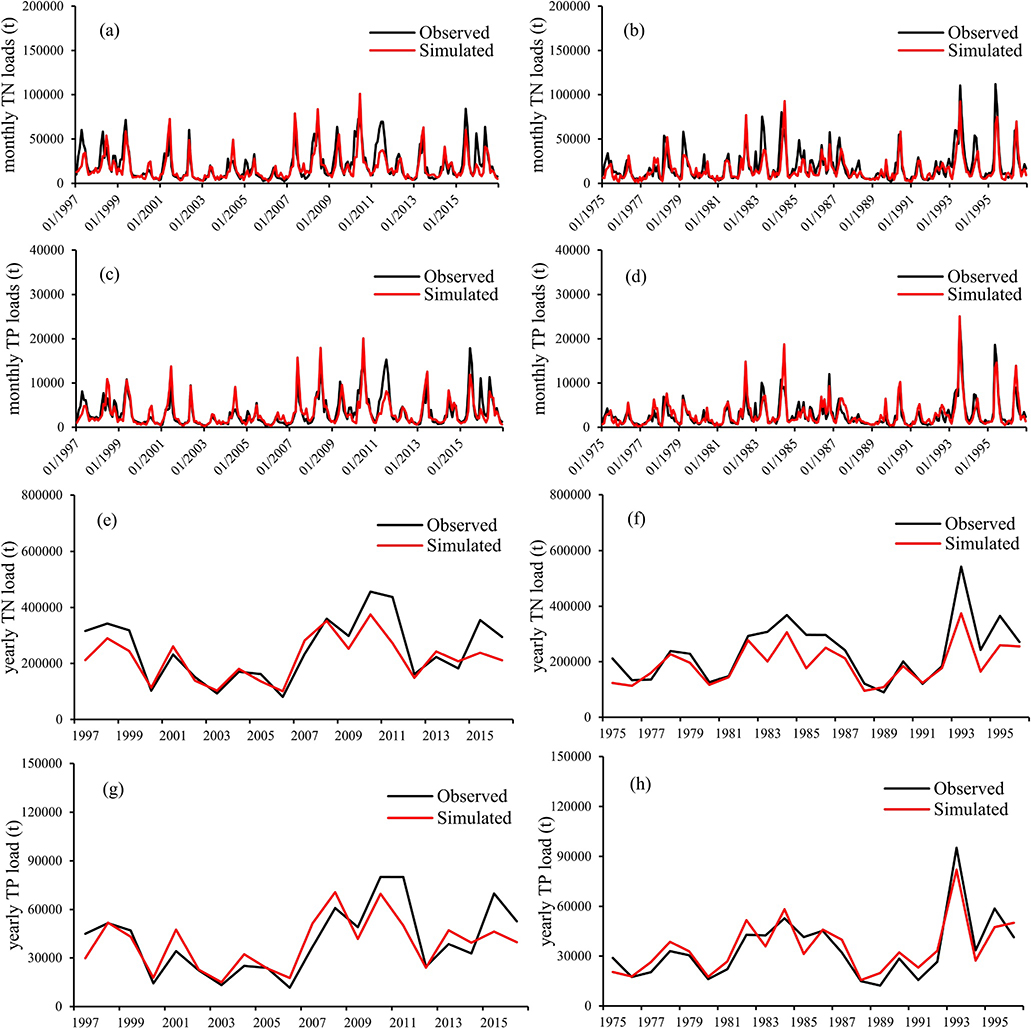
Monthly and annual simulated versus observed (a and b, e and f) total nitrogen and (c and d, g and h) total phosphorus comparison between Soil and Water Assessment Tool (SWAT) simulated and monitored at Hermann station during calibration period (1997–2016) and validation period (1975–1996).

The goal for large-scale simulations of sediment and nutrients is to ensure predictions replicate observations within an acceptable range, rather than to produce a perfect monthly or multi-year reproduction (Panagopoulos et al., 2015). Thus, PBIAS was usually used as the primary index to evaluate the model’s performance on sediment and nutrients. The PBIAS index of sediment was less than 30%, and that of TN and TP was less than 40%, at all the stations during the calibration and validation periods. This indicated the prediction results of sediment and nutrients were acceptable. In addition, the SWAT model performed very well for nutrients at the MORB outlet (Hermann station) (Figures 4a–4f), better than the other two stations (Sidney and Desoto) (Figure S3 and S4), showing that even with some positive or negative deviations at a local scale, the magnitude of TN and TP loads for the entire basin could still achieve good performance. Furthermore, most of the NSE and *R*^2^ values were greater than 0.5, indicating SWAT adequately captured the trends of the measured nutrient data. The final water quality parameter values were all within SWAT allowable ranges (Table S3 in the supporting information). These water quality parameter values did not vary among different gauge stations.

### 3.2. Water Quality Pattern of the Baseline Scenario

**Figure 5.**
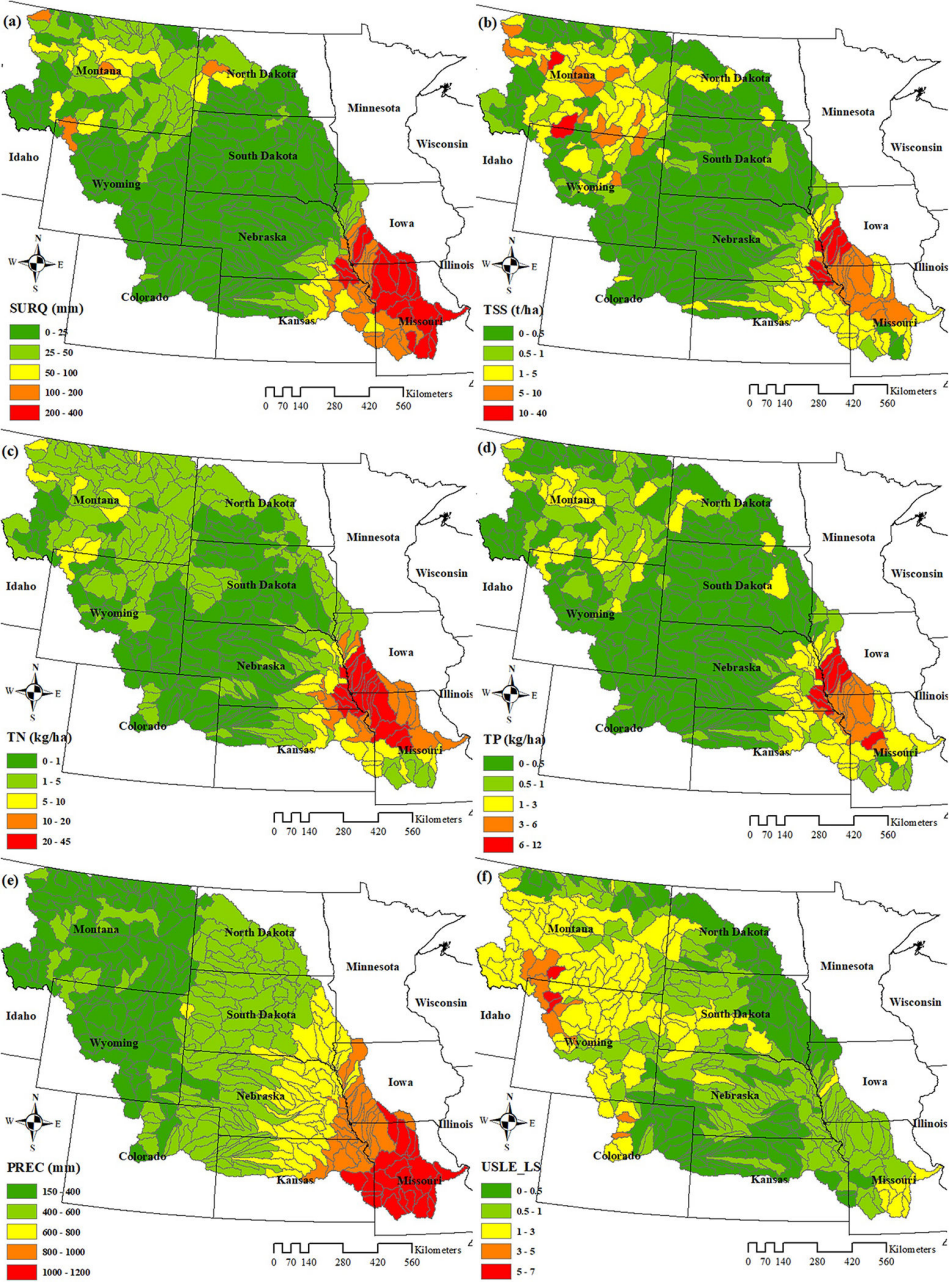
Spatial distributions under the baseline scenario for annual average (a) surface runoff, (b) total suspended sediment, (c) total nitrogen, (d) total phosphorus, (e) precipitation and (f) Universal Soil Loss Equation slope factor.

Spatial distribution of SWAT simulated annual average SURQ, total suspended sediment (TSS), and nutrients were examined to identify critical areas already producing high TSS and nutrient loads even before land conversion (Figures 5a–5d). The southeastern part of the MORB, especially the state of MO, produced SURQ greater than 200 mm in most of HUC-8 subbasins, higher than in other areas of the basin (Figure 5a). This was mainly due to precipitation (PREC) distribution within the MORB, with more precipitation in the southeast and less in the northwest (Figure 5e). High sediment producing areas (TSS ≥ 1.0 tons/ha) were primarily concentrated in two regions (Figure 5b): the upper and lower MORB. The high TSS from the upper MORB was partly due to the exceptionally rugged topography in that area, which was presented in the spatial pattern of the topographic factor (USLE_LE) (Figure 5f). The USLE_LE is a comprehensive topographic parameter used in the SWAT model which reflects the effects of slope and slope length (Neitsch et al., 2011). The larger this parameter, the greater TSS the area produces. By contrast, the high TSS production from the lower MORB was due to the combination of relatively high precipitation and the large crop acreage present in this region (Figures 2 and 5e).

As expected, high sediment producing areas in the lower MORB also produced high TN (>10 kg of N/ha) and TP yields (>3 kg of P/ha) (Figures 5c and 5d), as shown in other MORB studies (Wu and Zhang, 2015). Two factors were related to such a spatial pattern. The first factor was precipitation, as most of the lower MORB region had an annual precipitation rate greater than 800 mm, which meant that more nutrients would be transported due to high SURQ and sediment loss. The other factor was agricultural activity, a major source of N and P loadings (Cibin et al., 2012). This could be observed in the correspondence of nutrient hotspots with more agricultural regions, such as in IA.

Since the primary concern of this study is nutrient loading, the top 10 HUC-8 subbasins which produced the greatest nutrient loads were also examined (Table 9), mainly based on TN load. These subbasins all had annual precipitation amounts higher than 800 mm and cropland percentages larger than 37% (Table 9), which further supported the notion that precipitation and agricultural activity were two critical factors that determined the spatial pattern of nutrient loss. Notably, however, neither the highest amount of precipitation nor cropland percentage corresponded to the subbasin 10240009 in IA with the largest TN load, suggesting TN was affected by a combination of these factors and not one over the other. In addition, the ranking of SURQ, sediment, and TP of these HUC-8 subbasins were not all consistent with the influencing factors mentioned above, such as precipitation, topography, and agricultural activity. This further demonstrated that they were affected by a combination of these factors. For example, for most of these subbasins, the greater the precipitation, the greater the SURQ. However, there were still some exceptions, such as subbasins 10240009 and 10240003 in IA. Although the annual precipitation rate of the former was larger, its SURQ was still smaller because of smaller cropland percentage compared to the latter. Similarly, the precipitation of subbasin 10300104 in MO was the largest, while its sediment yield was not very large (9.8 t/ha) relative to other subbasins (Table 9), most likely due to a relatively small USLE_LS factor (0.5). The USLE_LS value of 10230006 in IA and NE ranked second while its sediment production was just 12.4 t/ha, due to the relatively small cropland percentage (48.1%). These same rules also applied to TN and TP. Although their spatial pattern at the regional basin scale was determined by precipitation and agricultural activity, the spatial distribution at the HUC-8 subbasin-scale was controlled by their combined effects.

### 3.3. Impact of Land Use Change on Water Quality

#### 3.3.1. Nutrient Loading Changes at the Outlet

**Figure 6.**
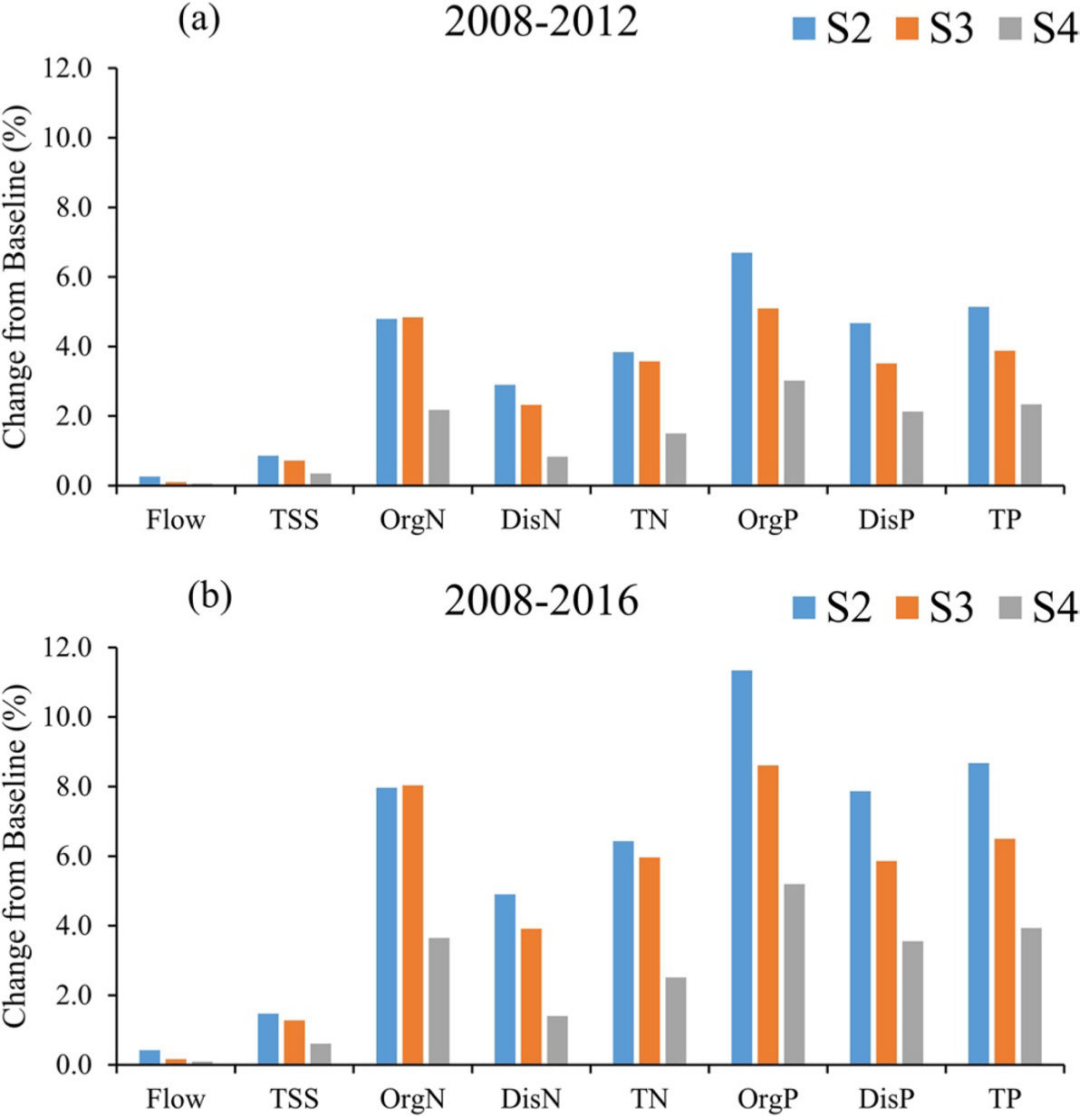
Mean Annual Changes in flow, total suspended sediment, organic nitrogen (including organic and ammonium nitrogen), dissolved nitrogen (including nitrate and nitrite), total nitrogen, organic phosphorus, dissolved phosphorus (refers to mineral phosphorus), and total phosphorus loads between the baseline scenario and different biofuel scenarios (S2, S3, S4) during (a) 2008–2012 and (b) 2008–2016.

The annual average streamflow, TN, and TP of the MORB were 2581.3 m^3^/s, 213.6 × 10^3^ t/yr and 39.1 × 10^3^ t/yr, respectively (Table 10). The cropping scenarios generally did not affect water quantity (Table 10; Figure 6). For example, annual flow only increased by 6.7 m^3^/s (0.26% of the baseline scenario) for the continuous corn scenario. The main reason was the area converted from noncrop land to crop land was relatively low (0.77% of the total area of MORB). The increase in sediments from the cropping scenarios was also very low compared to the results from baseline: just 0.86% for the continuous corn scenario. By contrast, change in nutrient loading was much greater than that of flow and sediment. For example, TN and TP increased 3.8% and 5.1% for the continuous corn scenario, respectively. Overall, TN increased 1.5%–3.8% (3,200–8,200 t/yr) during the 2008–2012 period and 2.5%–6.4% (5,400–13,800 t/yr) during the 2008–2016 period across different scenarios (Figure 6). The percentage change of TP from different scenarios was slightly higher than that of TN: 2.3%–5.1% (900–2,000 t/yr) during the period from 2008 to 2012 and 3.9%–8.7% (1,500–3,400 t/yr) during the period from 2008 to 2016 (Figure 6).

Comparing scenarios, continuous corn and corn/soybean rotations generally resulted in higher TN loads relative to the corn/wheat rotation. This was due to higher streamflow and sediment loss of the first two scenarios compared with the last. Corn and soybean have a larger USLE_C factor and CN value than that of winter wheat. A lower CN value would generate lower surface flow, while a lower USLE_C value indicates a lower erosion potential. In addition, winter wheat can act as protection from soil erosion. The difference in TN between continuous corn and corn/soybean rotation scenarios was quite small, a result also found in Deb et al.’s study in the Upper Mississippi River Basin (2015). They indicated that replacing corn-soybean rotations with continuous corn did not result in any significant change in TN load (Deb et al., 2015). Dissolved N (nitrate and nitrite) loading from the continuous corn scenario was greater than that from the corn/soybean rotation scenario (Figure 6), due to a greater fertilizer application rate required by corn (141.0 kg/ha) than by soybean (4.5 661 kg/ha); while the organic N (OrgN) loading from the former was slightly smaller (Figure 6), possibly due to a higher OrgN content in residue of soybean than corn. Thus the difference in TN between these two scenarios was small. The TSS loads from these two scenarios were similar based on the attributes of both crops used in SWAT (USLE_C factor, CN values).

Total P loading from the continuous corn scenario was larger than that from the other two cropping scenarios. This was because dissolved P, part of which came from fertilizer, was the main component of the TP (Table 10), and the P fertilizer application rate for corn was larger than those for soybean and wheat (Table 2). Comparing corn/soybean with corn/wheat rotation scenarios, the TP from the corn/soybean rotation scenario was larger than that from the corn/wheat rotation scenario although the P fertilizer requirement of soybean was lower than that of wheat. This was probably due to higher streamflow from the corn/soybean rotation than that from the corn/wheat scenario. Overall, continuous corn had the most adverse effect on water quality compared to the corn/soybean and corn/wheat rotations, an observation which had also been shown in other studies (Gu et al., 2015).

#### 3.3.2. Water Quality Change at the Subbasin Scale

##### 3.3.2.1. Change in Unit Area at Subbasin Scale

**Figure 7.**
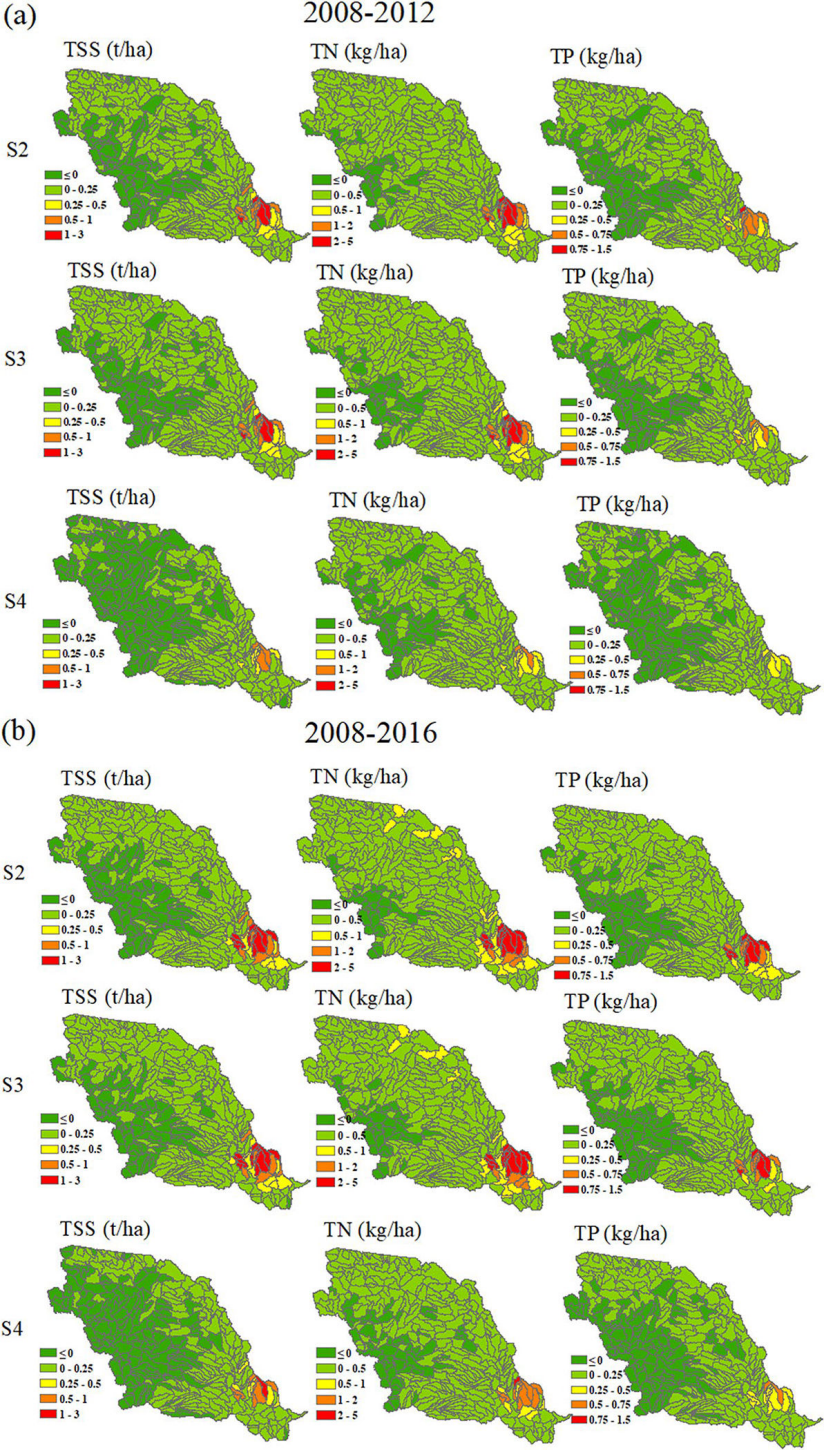
Differences in per unit area (refer to per hectare of land area) of total suspended sediment, total nitrogen and total phosphorus at S2 (baseline vs. continuous corn), S3 (baseline vs. corn/soybean) and S4 (baseline vs. corn/wheat) during (a) 2008–2012 and (b) 2008–2016.

On average, increases of TSS, TN and TP per hectare of land area across the entire basin were relatively low (Figure 7a). For example, increases of sediment per unit area of land use were 0.05, 0.05, and 0.02 t/ha, those of TN were 0.13, 0.12, and 0.05 kg/ha, and those of TP were 0.03, 0.03, and 0.01 kg/ha among different conversion scenarios and baseline, respectively. However, their spatial patterns showed unevenness and some regions showed relatively high changes because of the heterogeneity of land use change. In general, the areas with the greatest change corresponded to the highest pollutant producing areas in the baseline scenario, located in the lower MORB (Figures 5c, 5d, and 7) (e.g., IA, MO, NE, and KS). The top four HUC-8s with the greatest changes in TN between the continuous corn and baseline scenarios were 10240009 in IA, 10280102 in IA and MO, 10240007 in KS and NE, and 1028010 in IA and MO (Table 11); and these subbasins also exhibited the largest sediment changes between these two scenarios, which further proved the rule mentioned above that high sediment producing areas often produced high TN, as the organic form of N attached to soil particles was transported with sediment. Furthermore, they were also the top four subbasins with the greatest changes in TP. This pattern was also found in other studies, indicating that TP followed a very similar pattern to sediment due to its strong association with sediment (Neitsch et al., 2011; Panagopoulos et al., 2017). However, the rankings of TP and TN were not exactly one-to-one, likely because organic phosphorus transported with sediment represented a major portion of TP, while dissolved nitrogen moving with surface water accounted for a considerable proportion of TN (Table 9).

In general, the spatial distribution of changes in water quality depended more on basin characteristics, such as high precipitation rates at the southern part of MORB, than the individual cropping scenario. The top 10 subbasins with the highest TN change from 2008 to 2012 of continuous corn (Table 11) were also the top 10 for the other two scenarios, indicating different scenarios did not change the general spatial pattern of the TN variation from the baseline. However, different crops and rotations from different scenarios did lead to small variations in TN at the margins. For example, the TN ranking of HUCs 10240007 and 10280101 were switched between the continuous corn and the corn/wheat rotation. Similarly, the comparison between different scenarios involving sediment and TP also showed the same phenomenon as that of TN. Overall, HUCs 10240009, 10280102, 10280101, and 10240007 located at IA, MO, NE, and KS were the hotspots with the greatest increases in sediment and nutrient loadings (Table 11).

Spatial variations of differences per hectare of conversion of TSS, TN, and TP between the different cropping scenarios and the baseline were also analyzed, as they showed the most susceptible areas impacted by crop conversion (Figure S5a in supporting information). Of particular interest was that their spatial patterns were very similar to those of the baseline scenario (Figures 5b–5d). This indicated that the vulnerable areas in general corresponded to the highest pollutant producing areas in the baseline. For example, five of the top 10 HUC8s (10240009 in IA, 10240006 in NE, 10240008 in KS and NE, 10240010 and 10240012 in IA and MO) producing the highest nutrient loads (Table 9) were in Table 11. Although the other five (10230007 and 10240002 in IA, 10300104 in MO, 10230006 in IA and NE, and 10240003 in IA) in Table 9 were not listed in Table 11 (top 10 HUC8s with the largest sediment and nutrient changes) due to relatively small crop expansion rate (less than 1.2%), they were also important because of the high nutrient loadings they had already produced in baseline.

##### 3.3.2.2. Change in Percentage at Subbasin Scale

**Figure 8.**
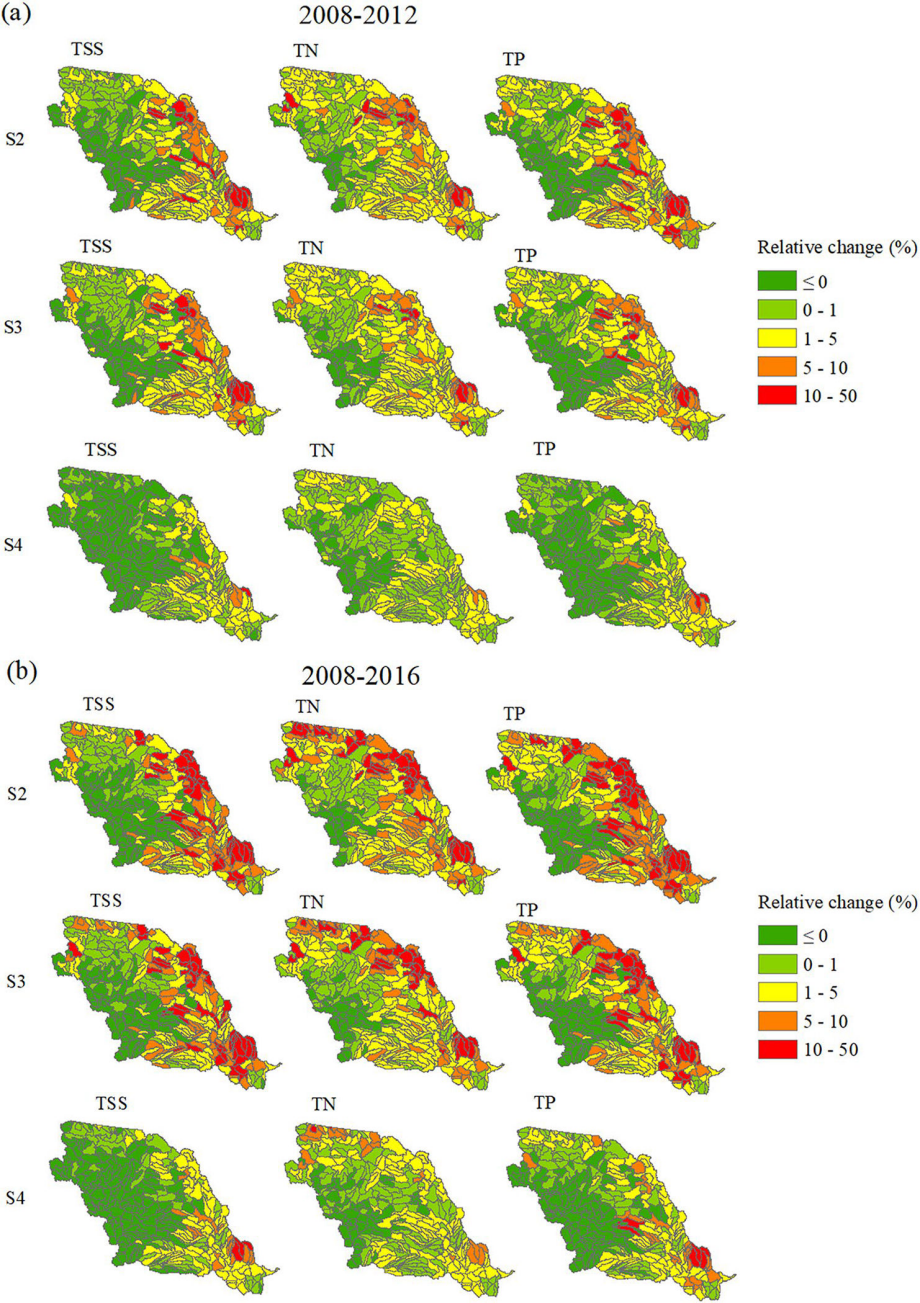
Percent differences relative to baseline for total suspended sediment, total nitrogen and total phosphorus for S2 (baseline vs. continuous corn), S3 (baseline vs. corn/soybean) and S4 (baseline vs. corn/wheat) during (a) 2008–2012 and (b) 2008–2016.

To identify the HUC8s with the highest relative water quality changes, percent changes in TSS (t), TN, and TP (kg) between the different cropping scenarios and the baseline were also analyzed for the 2008–2012 period (Figure 8). The highest percentage increases in TSS, TN, and TP loadings were not solely confined to those subbasins located in the lower MORB, corresponding to the absolute changes reported above (Figure 7). Subbasins located in ND and SD had the highest percentage increases, corresponding to the areas with the highest percentage of noncrop land to crop land conversion (Figure 2). However, those areas with the highest percentage increases located in ND and SD did not produce the highest absolute total loads; rather, some produced relatively low absolute total loads. For example, HUC8 10160004 in ND and SD produced 0.134 kg/ha of TN for the baseline scenario, and 0.152 kg/ha of TN for the continuous corn scenario even though the TN was increased by 13% from the baseline. For some subbasins, high percentage changes resulted from the low total loading in the baseline (low agricultural percentage). In other words, a large percentage increase of a small starting value could still yield a relatively small absolute value.

#### 3.3.3. Water Quality Change During Different Periods

The patterns of water quality changes that occurred at the outlet of the MORB were similar across our two study focus periods of 2008–2012 and 2008–2016 (Figure 6). The changes during the period from 2008 to 2016 were about 1.5 times greater than those that occurred between 2008 and 2012 (Figure 6), reflecting continued cropland expansion from 2012 to 2016. Noncropland to cropland conversion was 0.77% of the total basin area for the period from 2008 to 2012, and 1.18% for the period from 2008 to 2016. Therefore, the adverse impact on water quality continued to increase due to the on-going cropland expansion during this overall time period. The spatial variation in water quality changes between different cropping scenarios and the baseline in per unit area change and percent change during the 2008–2016 period was also very similar to that of 2008–2012, except that more basins across the MORB experienced higher percentage increases in loadings (Figures 7b and 8b, Figure S5b). In absolute loads, basins in the lower MORB, especially in IA, MO, NE, and KS, continued to be the hotspots of water quality degradation. Those “hotspots” should be avoided for further crop conversion and targeted first to achieve greater water quality benefits.

#### 3.3.4. Water Quality Effects of Crop Expansion From Previous Studies

A number of studies have attempted to evaluate the changes of water quantity and quality in response to crop expansion (Deb et al., 2015; Gu et al., 2015; Panagopoulos et al, 2014, 2017; Wu and Zhang, 2015; Yu and McCarl, 2018). Panagopoulos et al., (2014) evaluated the potential effects of continuous corn expansion across the Upper Mississippi River Basin (UMORB) on water quality and indicated that increasing continuous corn would result in increased N loss to water bodies. Yu and McCarl (2018) analyzed the effect of land use change on water quantity and quality using a land use model, FASOM-GHG, and their results indicated that increases in crop area significantly degraded water quality of the MORB. Results from this study echoed these previous works, further proving that crop expansion would degrade water quality.

This study was unique in that it focused on the water quality effects of crop expansion based on observation of land use/land cover changes (from 2008 to 2012 and 2008 to 2016), whereas those in the literature were based on future hypothetical scenarios to help understand potential environmental impacts of crop expansion. As the settings of those hypothetical scenarios were likely different from real situations in many aspects, those results had limitations and might not accurately represent results. For example, the future land use changes used in those studies didn’t reflect spatial variation, and a uniform change such as 25% or 50% increase across the entire study area was usually applied (Deb et al., 2015; Panagopoulos et al., 2017). Thus, most of the studies discussed the pollutant loading changes at the outlet of the basin, but failed to discuss the spatial variation of water quality changes within the basin and identify “hotspots”. Since this study used observation of land use/land cover changes, which were heterogeneric, “hotspots,”—such as some subbasins in IA, MO, NE, and KS in the lower MORB—were identified. Furthermore, the actual change in area was relatively low, 0.77% from 2008 to 2012 (Lark et al., 2015) and 1.18% from 2008 to 2016 (Lark et al., 2020).

This study provides valuable information on the water quality loading changes of the MORB, water quality change metrics per unit area of land use, and “hotspots” or watersheds experiencing the greatest increase in nutrient loadings. Such information could be used to help prioritize areas for grassland conservation and inform agricultural policy (Lark, 2020) or help ensure proper decision making for watershed protection or watershed restoration projects. For example, this work may be linked with known state water quality criteria to address “so what” questions such as which watersheds are (a) under the criteria and may be pushed over the criteria, or (b) already over the threshold and getting worse, such that timely measures can be developed and implemented to prevent further water quality degradation.

## 4. Conclusions

Our study results suggest that cropland expansion onto grasslands degraded water quality in the US Midwest between 2008 and 2016. The greatest percentage increases of TN and TP loading occurred in ND and SD, coinciding with the highest amount of grassland conversion, yet these areas contributed small absolute amounts of TN and TP to the total basin loads. Instead, specific watersheds, or “hotspots,” in the lower MORB—especially in IA, MO, NE, and KS—contributed the greatest amounts of TN and TP to basin-wide loads. Grassland converted to continuous corn increased nutrient loadings the most, with the TN increased by 13,800 t/yr (6.4%) and TP increased by 3,400 t/yr (8.7%); whereas grassland converted to corn/wheat increased the nutrient loadings the least, with the TN increased by 5,400 t/yr (2.5%) and TP increased by 1,500 t/yr (3.9%). We anticipate that this information will be used by a variety of federal, state and local agencies with the goal of reducing water pollution. Our results suggest divergent management strategies depending upon objectives. Targeting “hotspots” in the lower MORB would likely help downstream water quality (e.g., reducing the hypoxic zone in the Gulf of the Mexico) the most; whereas interventions in watersheds with the highest percentage increases in TN and TP but still low with absolute total loads, such as those in the Dakotas, may help preserve conditions in less impacted watersheds.

## Supplementary Material

Supplement1

## Figures and Tables

**Table 1:** Summarized Information of the Input Data Used in the SWAT Model

Data-set	Description of data	Source of data

Elevation	90 meter digital elevation model	USGS (2016)
Soil	1:250,000 STATSGO soil map	USDA-NRCS (2013)
Land use	Assignment of crop rotations or other land use based on Cropland Data Layers data from 2008 to 2009	USDA-NASS (2016)
Weather	Historic daily precipitation, maximum temperatures, and minimum temperatures from 1,721 NWS stations	NCDC (2016)
Point sources	Regression of population and SPARROW model outputs	Schwarz et al. (2006)
Fertilizer input	Nitrogen and phosphorus rates applied in corn, soybean, spring wheat and winter wheat	USDA-NASS (2017)
Tillage practices	No-till, mulch till, reduced till, and conventional till practices	Baker (2011)
Reservoirs	Reservoirs with maximum storage larger than 25,000 acre feet	USACE (2012)

Abbreviation: SWAT, Soil and Water Assessment Tool.

**Table 2:** The Average Values of N and P Fertilizer Application Rates for Corn, Soybean and Spring Wheat and Winter Wheat at MORB

Crop type	Fertilizer type	Average rate (kg/ha)^a^

Corn	N	141.0
	P	39.9
Soybean	N	4.5
	P	12.3
Spring Wheat	N	64.4
	P	27.1
Winter Wheat	N	61.8
	P	22.2

Abbreviation: MORB, Missouri River Basin.

a State average of each crop for the MORB.

**Table 3:** List of Available Periods of Measured Streamflow, Total Suspended Sediment (TSS), Total Nitrogen (TN) and Total Phosphorus (TP) at 10 USGS Gauge

Site name	Site number	Hydrologically independent	Drainage (km^2^)	Streamflow	TSS	TN	TP

Culbertson	06185500	Yes	232,731	1975–2019	–	–	–
Sidney	06329500	Yes	178,966	1975–1990 2000–2019	1975–2012	1975–1990 2000–2019 (311)	1975–1990 2000–2019 (349)
Bismarck	06342500	No	482,776	1975–2019	–	–	–
Forestburg	06477000	Yes	45,617	1975–2019	–	–	–
Ashland	06801000	Yes	216,524	1988–2019	–	–	–
Sioux	06486000	No	814,814	1975–2019	1975–1976 1991–2000 2003–2019	–	–
Omaha	06610000	No	836,049	1975–2019	1975–1976 1991–2003 2008–2019	–	–
Desoto	06892350	Yes	154,768	1975–2019	–	1975–2019 (199)	1975–2019 (214)
Bagnell	06926000	Yes	36,260	1975–2019	–	–	–
Hermann	06934500	No	1,353,270	1975–2019	–	1975–2019 (463)	1975–2019 (468)

Abbreviations: USGS, US Geological Survey.

**Table 4:** Simulation Scenarios

No	Description	Notes

1	Areas identified as noncropland (10,154 km^2^) were assumed as grassland for the land use type, prior to their conversion to cropland (baseline)	The 2008 and 2009 CDLs were combined; then the combined layers were overlaid with locations of cropland conversion from Lark et al. (2015, 2020) to identify areas where noncropland was converted to cropland. For scenario 1, which is the baseline, those identified noncropland was assumed as grassland.
2	Grassland (10,154 km^2^) from scenario 1 was converted to continuous corn	
3	Grassland (10,154 km^2^) from scenario 1 was converted to corn/soybean rotation	
4	Grassland (10,154 km^2^) from scenario 1 was converted to corn/wheat rotation	

Abbreviation: CDLs, Cropland Data Layers.

**Table 5:** Monthly Streamflow Calibration and Validation Statistics

	Calibration (1997–2016)			Validation (1975–1996)		
Calibration points	*R*^2^	NSE	PBIAS	*R*^2^	NSE	PBIAS

Culbertson	0.81	0.51	−14.1	0.57	0.27	14.8
Sidney	0.75	0.67	−0.6	0.7	0.56	24.8
Bismarck	0.79	0.53	−10.2	0.75	0.55	14
Forestburg	0.52	0.5	24.6	0.39	0.26	−43.7
Ashland	0.51	−0.11	2.5	0.44	0.22	22.2
Sioux	0.81	0.75	−11.6	0.73	0.64	−0.8
Omaha	0.81	0.8	2.2	0.77	0.61	13.5
Desoto	0.6	0.52	1.4	0.77	0.74	14
Bagnell	0.85	0.85	4.2	0.89	0.88	1.2
Hermann	0.7	0.67	−3.6	0.75	0.74	6

Abbreviations: NSE, Nashe-Sutcliffe efficiency; PBIAS, Percent bias; *R*^2^, coefficient of determination.

**Table 6:** Annual Streamflow Calibration and Validation Statistics

	Calibration (1997–2016)			Validation (1975–1996)		
Calibration points	*R*^2^	NSE	PBIAS	*R*^2^	NSE	PBIAS

Culbertson	0.9	0.52	−13.7	0.66	0.29	14.9
Sidney	0.8	0.64	−1.1	0.82	−0.1	23.8
Bismarck	0.85	0.65	−10.2	0.85	0.44	14.2
Forestburg	0.63	0.57	24.4	0.49	0.29	−44.2
Ashland	0.57	−0.25	2.3	0.75	0.33	23
Sioux	0.85	0.77	−11.7	0.71	0.51	−0.9
Omaha	0.85	0.85	2	0.91	0.47	13.4
Desoto	0.82	0.8	1.2	0.93	0.89	13.7
Bagnell	0.94	0.92	4.3	0.98	0.96	1.6
Hermann	0.91	0.89	−3.6	0.98	0.95	5.9

Abbreviations: NSE, Nashe-Sutcliffe efficiency; PBIAS, Percent bias; *R*^2^, coefficient of determination.

**Table 7:** Monthly TSS, TN and TP Calibration and Validation Statistics

		Calibration (1997–2016)			Validation (1975–1996)		
Variable	Calibration points	*R*^2^	NSE	PBIAS	*R*^2^	NSE	PBIAS

TSS	Sidney	0.55	0.24	−13.6	0.57	0.56	21.1
	Sioux	0.56	0.45	−20.7	0.18	−0.31	11.6
	Omaha	0.4	−0.25	−20.3	0.3	0.12	27.9
TN	Sidney	0.6	−0.13	−28.8	0.68	0.6	29.2
	Desoto	0.59	0.33	−28.1	0.74	0.69	26.8
	Hermann	0.69	0.67	12.2	0.68	0.65	17.7
TP	Sidney	0.57	0.56	−4.6	0.59	0.53	27.9
	Desoto	0.65	0.55	2	0.84	0.83	13.8
	Hermann	0.71	0.7	3.9	0.78	0.75	−2.9

Abbreviations: NSE, Nashe-Sutcliffe efficiency; PBIAS, Percent bias; *R*^2^, coefficient of determination; TN, total nitrogen; TP, total phosphorus; TSS, total suspended sediment.

**Table 8:** Annual TSS, TN and TP Calibration and Validation Statistics

		Calibration (1997–2016)			Validation (1975–1996)		
Variable	Calibration points	*R*^2^	NSE	PBIAS	*R*^2^	NSE	PBIAS

TSS	Sidney	0.82	−0.15	−20.8	0.64	0.58	15.5
	Sioux	0.69	0.61	−22.9	0.31	0.26	2.5
	Omaha	0.49	−0.1	−22.2	0.42	−0.48	25
TN	Sidney	0.53	−0.39	−29.4	0.81	0.1	28.1
	Desoto	0.62	0.36	−28.1	0.89	0.67	26.8
	Hermann	0.76	0.65	12.2	0.84	0.62	17.7
TP	Sidney	0.56	0.53	−4.6	0.67	0.36	28.1
	Desoto	0.68	0.62	2	0.93	0.87	13.8
	Hermann	0.68	0.67	3.9	0.86	0.85	−2.9

Abbreviations: NSE, Nashe-Sutcliffe efficiency; PBIAS, Percent bias; *R*^2^, coefficient of determination; TN, total nitrogen; TP, total phosphorus; TSS, total suspended sediment.

**Table 9:** Information on the Top 10 HUC8 Subbasins Producing the Highest Nutrient Loads

HUC8	PREC (mm)	SurQ (mm)	USLE_LS	Cropland Percentage (%)	TSS (t/ha)	TN (kg/ha)	TP (kg/ha)	OrgN (kg/ha)	DisN^a^ (kg/ha)	OrgP	DisP^b^ (kg/ha)

10240009	931.6	198.7	0.8	49.5	16.5	42.0	8.3	36.6	5.4	8.2	0.1
10230007	838.4	191.0	1.1	69.2	30.1	41.8	10.7	33.6	8.2	10.5	0.1
10240006	801.2	215.3	0.6	60.8	17.5	40.1	8.8	31.6	8.5	8.7	0.1
10240002	895.0	212.4	0.8	76.5	19.2	38.9	9.9	30.9	8.0	9.7	0.2
10300104	1074.2	279.4	0.5	40.7	9.8	36.6	6.6	30.3	6.3	6.4	0.2
10240008	836.1	253.6	0.6	52.1	16.8	36.2	7.8	28.6	7.6	7.6	0.1
10230006	827.8	177.3	1.0	48.1	12.4	35.9	7.4	28.4	7.5	7.3	0.2
10240003	923.9	225.8	0.8	66.0	16.6	32.6	8.3	25.3	7.3	8.2	0.2
10240010	924.8	181.8	0.7	48.6	7.5	31.5	5.8	25.8	5.7	10.5	0.1
10240012	986.0	219.1	0.6	37.9	8.6	28.0	5.1	22.7	5.3	8.7	0.1

Abbreviations: DisN, dissolved nitrogen; DisP, dissolved phosphorus; OrgN, organic N; OrgP, organic phosphorus; PREC, precipitation; SURQ, surface runoff; TN, total nitrogen; TP, total phosphorus; TSS, total suspended sediment; USLE, Universal Soil Loss Equation; USLE_LS, USLE equation slope factor.

a Refers to nitrate and nitrite.

b Refers to mineral P.

**Table 10:** Mean Annual (1987–2016) SWAT Estimates of Flow, Sediment, N and P Constituents at the Outlet of the MORB Under Different Conversion Scenarios (2008–2012)

Scenario			1, 000 t/yr						
No	m^3^/s	Flow	TSS	OrgN	DisN^a^	TN	OrgP	DisP^b^	TP

1	Baseline	2581.3	35,860.3	106.2	107.4	213.6	9.0	30.1	39.1
2	Converted to continuous corn	2588.0	36,168.0	111.2	110.6	221.8	9.7	31.4	41.1
3	Converted to corn/soybean rotation	2583.8	36,118.7	111.4	109.9	221.3	9.5	31.1	40.6
4	Converted to corn/wheat rotation	2582.9	35,983.7	108.5	108.3	216.8	9.3	30.7	40.0

Abbreviations: DisN, dissolved nitrogen; DisP, dissolved phosphorus; MORB, Missouri River Basin; OrgN, organic N; OrgP, organic phosphorus; TN, total nitrogen; TP, total phosphorus; TSS, total suspended sediment.

a Refers to nitrate and nitrite.

b Refers to mineral P.

**Table 11:** The Top 10 HUC8 Subbasins With the Greatest Increase Per Unit (Hectare of Land Area) of TSS, TN and TP at S2 (Baseline VS. Continuous Corn), S3 (Baseline VS. Corn/Soybean) and S4 (Baseline VS. Corn/Wheat) During 2008–2012

	TSS (t/ha)				TN (kg/ha)				TP (kg/ha)		
HUC8^a^	S2	S3	S4	HUC8^a^	S2	S3	S4	HUC8^a^	S2	S3	S4

10240009	1.51	1.61	0.62	10240009	3.09	3.18	1.30	10240009	0.77	0.65	0.35
10280102	1.43	1.58	0.66	10280102	2.58	2.69	1.19	10280102	0.74	0.59	0.38
10280101	1.24	1.36	0.51	10240007	2.42	2.35	0.87	10280101	0.64	0.49	0.30
10240007	1.08	1.21	0.36	10280101	2.16	2.13	0.91	10240007	0.61	0.54	0.24
10280201	0.85	0.94	0.40	10240012	2.09	2.07	0.84	10280201	0.54	0.40	0.26
10240012	0.84	0.90	0.34	10240010	2.06	2.17	0.88	10240012	0.52	0.42	0.23
10240010	0.77	0.78	0.34	10280201	1.99	1.94	0.78	10240013	0.51	0.44	0.24
10240013	0.73	0.70	0.26	10240013	1.92	1.81	0.68	10240010	0.50	0.43	0.23
10240008	0.62	0.67	0.24	10280103	1.26	1.22	0.53	10240008	0.35	0.28	0.16
10240006	0.53	0.58	0.24	10240008	1.22	1.13	0.49	10280103	0.32	0.25	0.16

Abbreviations: TN, total nitrogen; TP, total phosphorus; TSS, total suspended sediment.

a All these HUC8 subbasins had land-use changes from grass to crop.

## Data Availability

The elevation data for this study is obtained from the USGS website at http://ned.usgs.gov/. Soil data is obtained from the USDA website at https://sdmdataaccess.nrcs.usda.gov/. The USDA Crop Data Layer data is obtained from the website at https://nassgeodata.gmu.edu/CropScape/. Weather data is obtained from US National Climatic Data Center at http://www.ncdc.noaa.gov/. Point source data is obtained from Hydrologic and Water Quality System (HAWQS) database at https://hawqs.tamu.edu/. Fertilizer data is obtained from the USDA-NASS Census of Agriculture at https://www.nass.usda.gov/AgCensus/. Tillage data is obtained from the website at https://pubs.usgs.gov/ds/ds573/. Reservoir data is obtained from the USACE national inventory of dams (available at https://nid.sec.usace.army.mil/ords/f?p=105:22:12281907402981::NO:::). USGS gauge measurements of streamflow and water quality are obtained from the USGS National Water Information System at https://waterdata.usgs.gov/nwis/sw

## Key Points:

- Recent crop expansion on water quality was assessed for the Missouri River Basin experiencing the highest rate of conversion of grasslands
- Converting to continuous corn had the most adverse effect on water quality, followed by corn/soybean rotation, and then corn/wheat rotation
- Watersheds in Iowa, Missouri, Nebraska and Kansas continue to be the “Hotspots” contributing the greatest amounts of nutrient loads
