# Supplementary material for: Assessing the Impacts of Recent Crop Expansion on Water Quality in the Missouri River Basin Using the Soil and Water Assessment Tool
Source: J Adv Model Earth Syst. Author manuscript; Available in PMC 2022 May 28. (PMC8318093; doi:10.1029/2020ms002284)
Supplement: Supplement1 [file NIHMS1715213-supplement-Supplement1.docx]

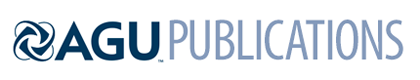


*Journal of Advances in Modeling Earth Systems*

Supporting Information for

**Assessing Recent Crop Expansion on Water Quality in the Missouri River Basin Using the SWAT Model**

Pan Chen^1,2,3^, Yongping Yuan^4^, Wenhong Li^2,3^, Stephen LeDuc^4^, Tyler Lark^5^, Xuesong Zhang^6^, and Christopher Clark^7^

^1^College of Water Resources Science and Engineering, Taiyuan University of Technology, Taiyuan 030024, China

^2^Oak Ridge Institute for Science and Education (ORISE), USEPA, Research Triangle Park, NC 27711, USA

^3^Earth and Ocean Sciences, Nicholas School of the Environment, Duke University, NC 27708, USA

^4^USEPA-Office of Research and Development, Research Triangle Park, NC 27711, USA

^5^University of Wisconsin Madison- Nelson Institute Center for Sustainability and the Global Environment, Madison, WI 53726, USA

^6^US Department of Energy-Pacific Northwest National Laboratory, College Park, MD 20740, USA

^7^USEPA-Office of Research and Development, Washington DC 20004, USA

**Contents of this file**

Figures S1 to S5

Tables S1 to S3


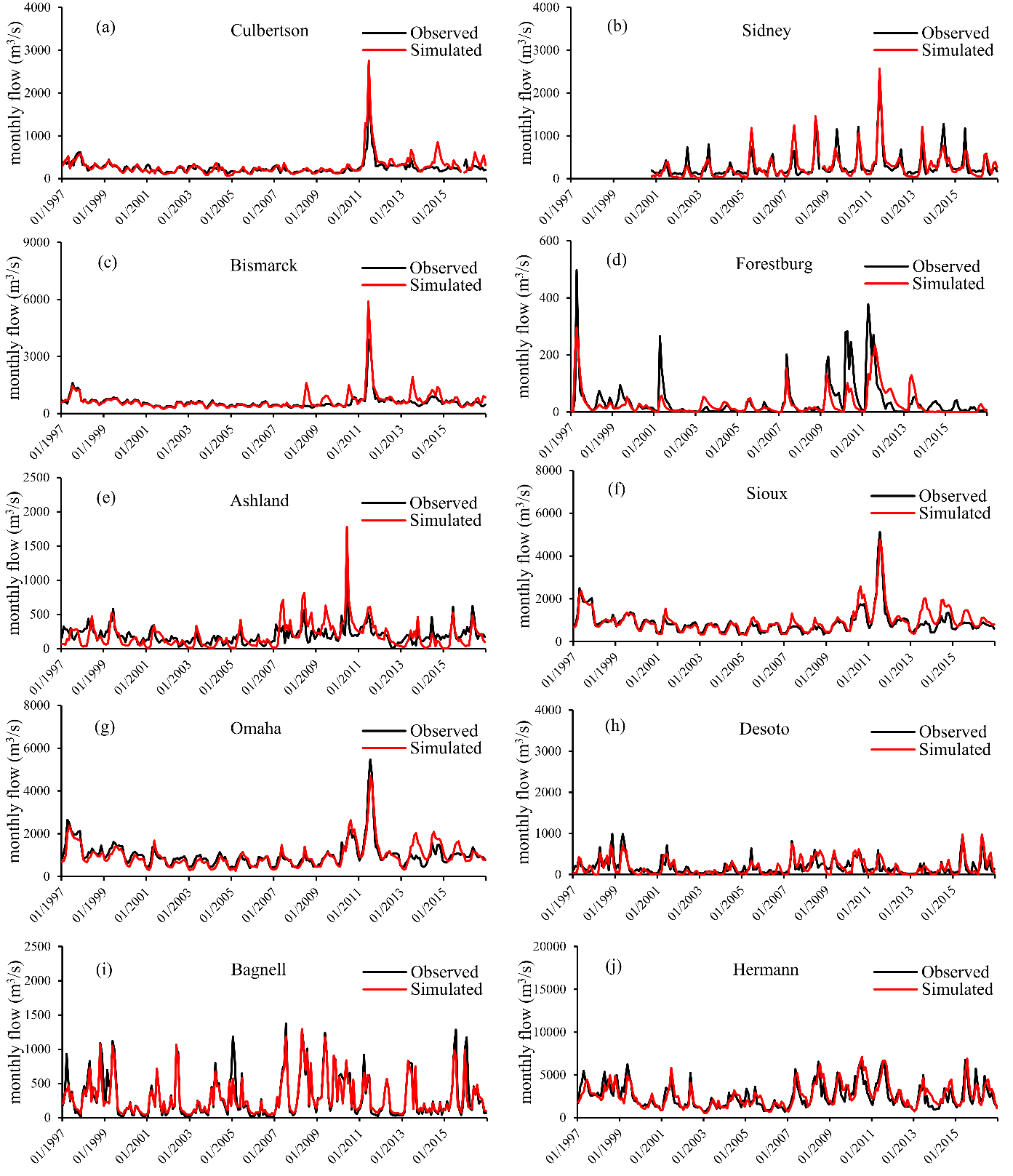


**Figure S1.** Monthly simulated versus observed flows during calibration period (1997-2016) at MORB


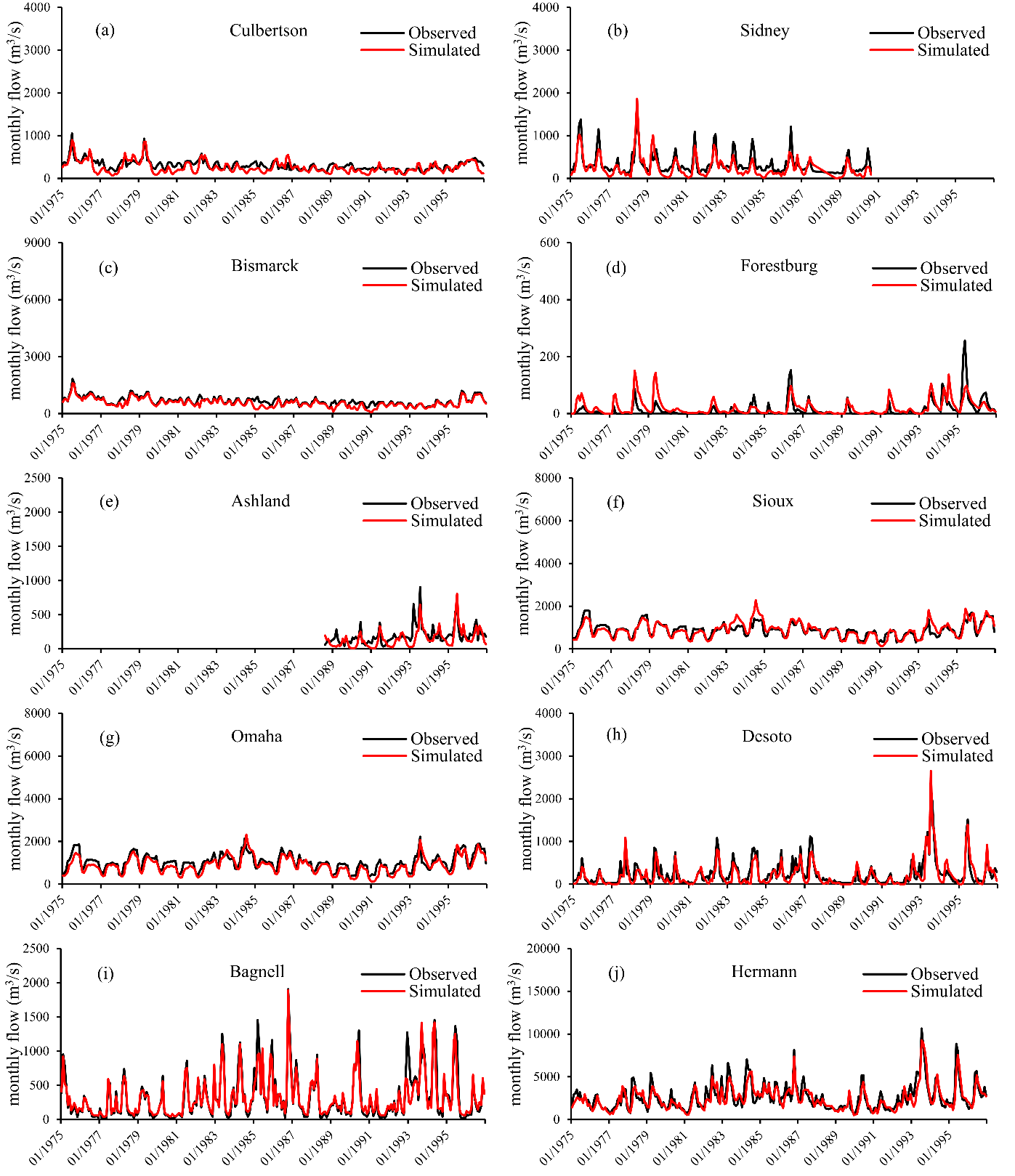
 **Figure S2.**  Monthly simulated versus observed flows during validation period (1975-1996) at MORB


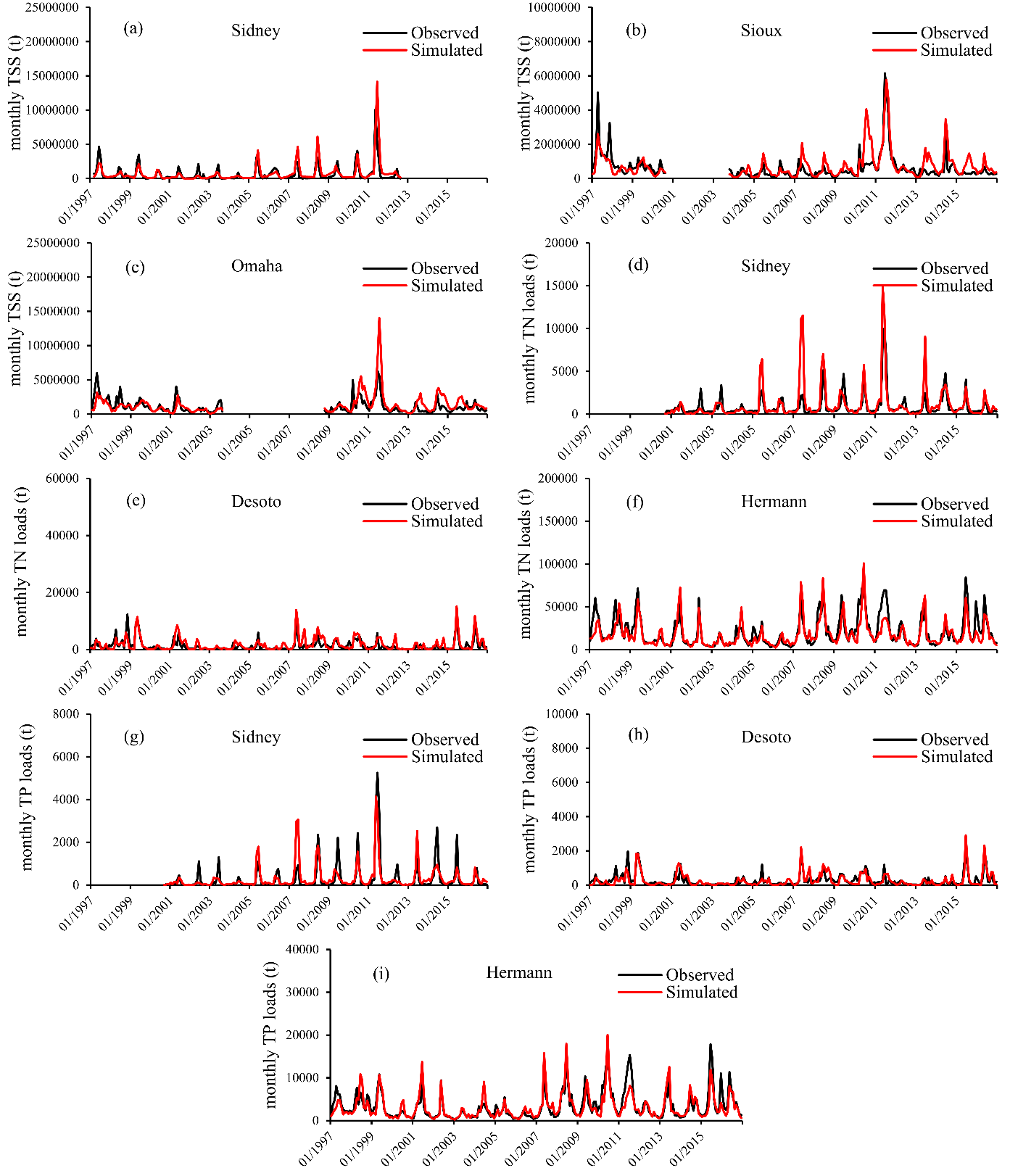


**Figure S3.** Monthly simulated versus observed TSS, TN and TP during calibration period (1997-2016) at MORB


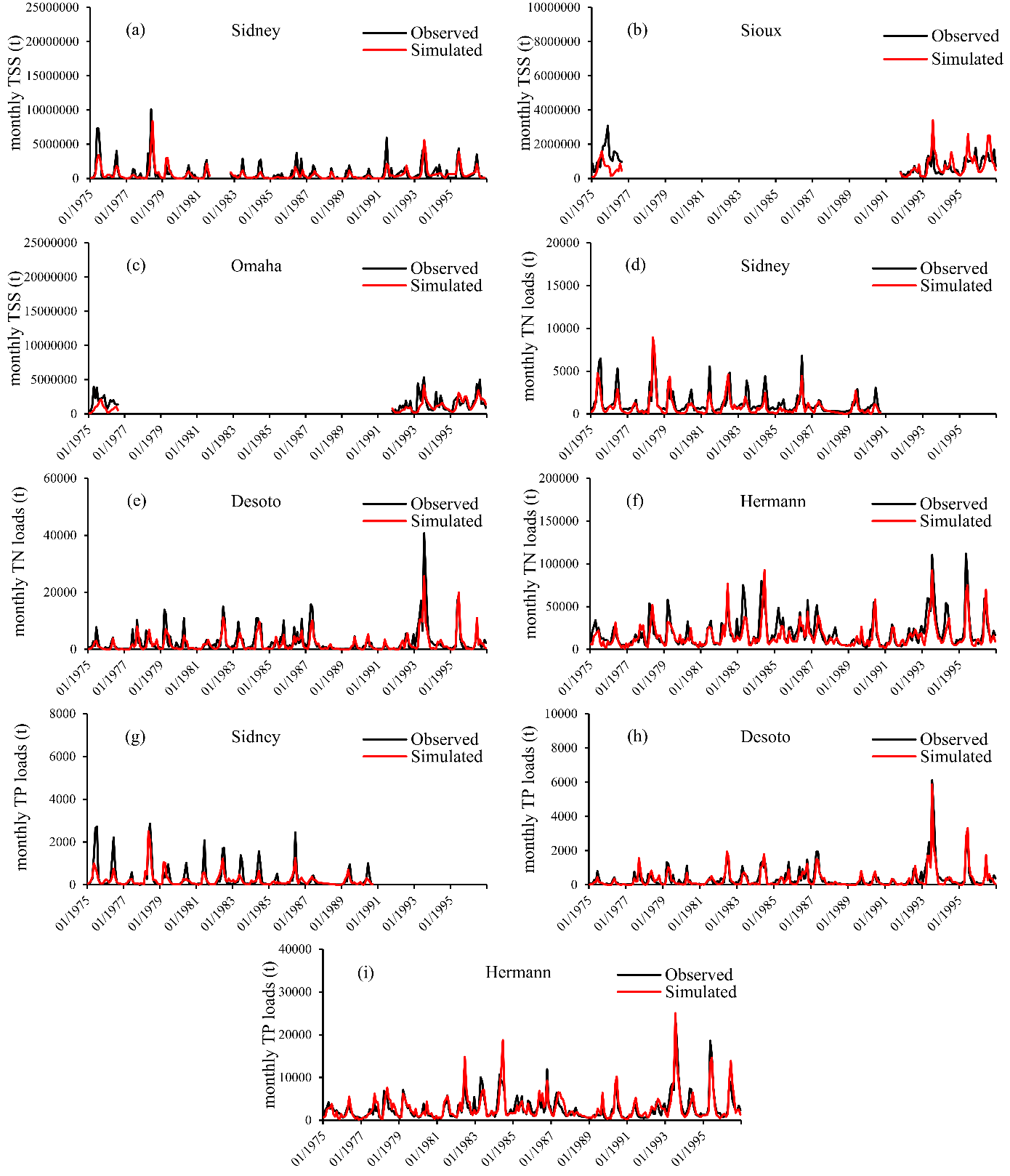
 **Figure S4.** Monthly simulated versus observed TSS, TN and TP during validation period (1975-1996) at MORB


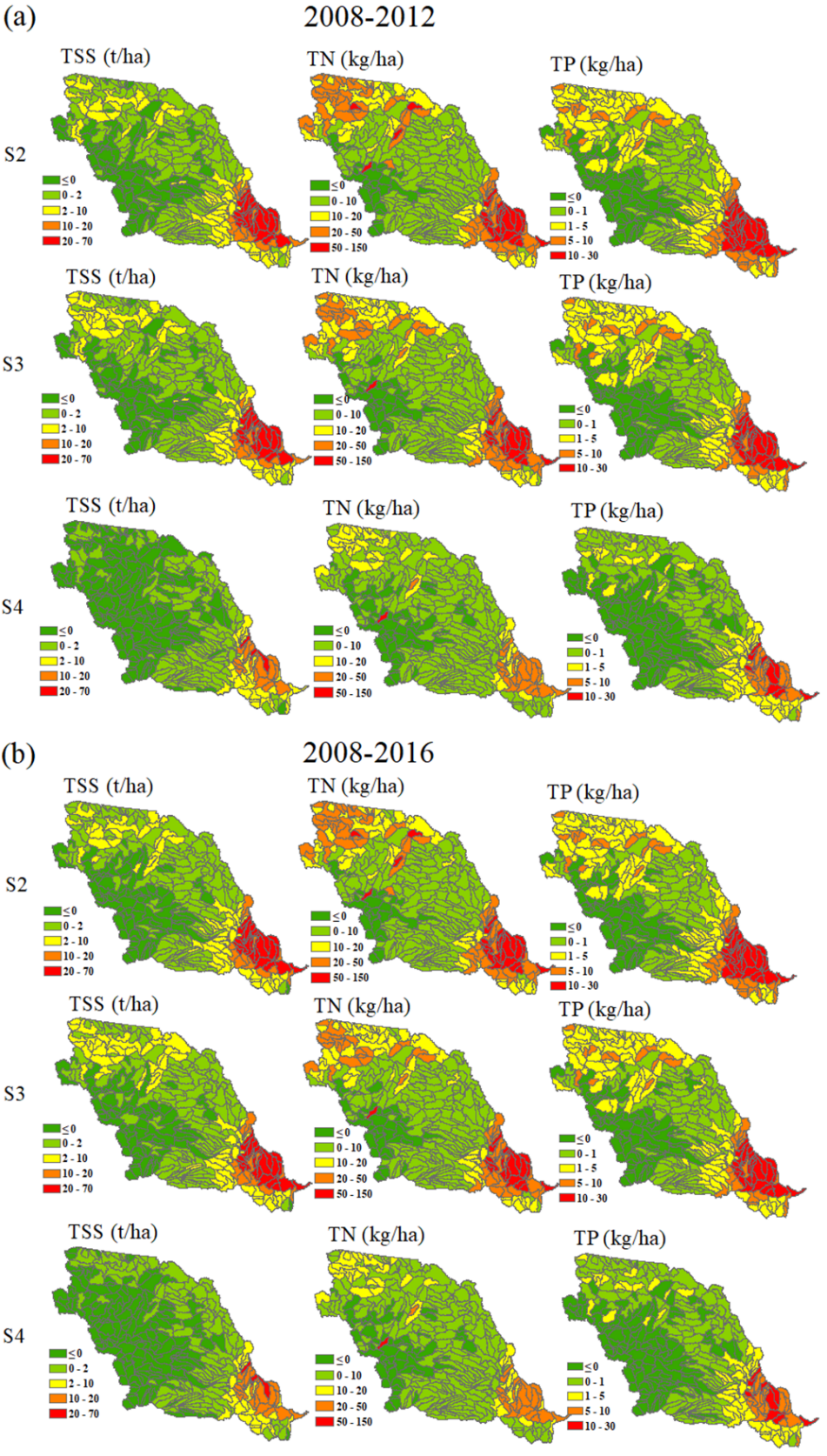


**Figure S5.** Differences in per unit area (refer to per hectare of conversion) of TSS, TN and TP at S2 (baseline vs. continuous corn), S3 (baseline vs. corn/soybean) and S4 (baseline vs. corn/wheat) during 2008-2012 (a) and 2008 to 2016 (b)

**Table S1.** Final crop rotation used in the SWAT model

| NO | Value | Land use of 2008 | Land use of 2009 | Percentage (%) | Cumulative percentage (%) | Final  crop rotation |
| --- | --- | --- | --- | --- | --- | --- |
| 1 | 469 | Corn | Soybean | 16.15 | 16.15 | CSOY |
| 2 | 454 | Soybean | Corn | 15.75 | 31.90 | SOYC |
| 3 | 301 | Corn | Corn | 8.37 | 40.27 | CORN |
| 4 | 302 | Fallow | Winter wheat | 8.03 | 48.31 | FAWW |
| 5 | 303 | Winter wheat | Fallow | 5.68 | 53.99 | WWFA |
| 6 | 306 | Spring wheat | Spring wheat | 4.20 | 58.19 | SWHT |
| 7 | 304 | Alfalfa | Alfalfa | 3.77 | 61.97 | ALFA |
| 8 | 307 | Alfalfa | Non-agriculture | 3.45 | 65.42 | ALFA |
| 9 | 562 | Soybean | Soybean | 3.36 | 68.78 | SOYB |
| 10 | 305 | Winter wheat | Winter wheat | 2.95 | 71.73 | WWHT |
| 11 | 308 | Winter wheat | Corn | 2.37 | 74.10 | WHTC |
| 12 | 318 | Durum Wheat | Durum Wheat | 1.84 | 75.93 | SWHT |
| 13 | 309 | Corn | Non-agriculture | 1.73 | 77.66 | CSOY |
| 14 | 310 | Spring wheat | Fallow | 1.49 | 79.15 | SWFA |
| 15 | 405 | Soybean | Non-agriculture | 1.46 | 80.61 | SOYB |
| 16 | 311 | Winter wheat | Non-agriculture | 1.34 | 81.96 | WWSY |
| 17 | 312 | Fallow | Non-agriculture | 1.24 | 83.20 | HAY |
| 18 | 313 | Fallow | Spring wheat | 1.18 | 84.37 | FASW |
| 19 | 316 | Winter wheat | Sorghum | 1.04 | 85.41 | WWHT |
| 20 | 314 | Corn | Fallow | 1.02 | 86.44 | CORN |
| 21 | 315 | Spring wheat | Non-agriculture | 0.98 | 87.41 | SWSY |
| 22 | 317 | Spring wheat | Winter wheat | 0.94 | 88.35 | SWHT |
| 23 | 490 | Soybean | Spring wheat | 0.94 | 89.29 | SYSW |
| 24 | 319 | Sunflower | Spring wheat | 0.92 | 90.21 | SFSW |
| 25 | 584 | Soybean | Winter wheat | 0.84 | 91.05 | SYWW |
| 26 | 320 | Sorghum | Fallow | 0.78 | 91.83 | SGHY |
| 27 | 321 | Spring wheat | Corn | 0.70 | 92.53 | SWCR |
| 28 | 322 | Fallow | Fallow | 0.65 | 93.18 | HAY |
| 29 | 537 | Winter wheat | Soybean | 0.62 | 93.80 | WWSY |
| 30 | 327 | Barley | Barley | 0.60 | 94.40 | BARL |
| 31 | 446 | Spring wheat | Soybean | 0.60 | 95.00 | SWSY |
| 32 | 632 | Sorghum | Soybean | 0.44 | 95.44 | SOYB |
| 33 | 323 | Winter wheat | Spring wheat | 0.42 | 95.86 | WWHT |
| 34 | 463 | Corn | Sunflower | 0.42 | 96.28 | CORN |
| 35 | 694 | Sorghum | Sorghum | 0.39 | 96.68 | SGHY |
| 36 | 324 | Corn | Winter wheat | 0.37 | 97.05 | CWHT |
| 37 | 423 | Spring wheat | Sunflower | 0.35 | 97.40 | SWSF |
| 38 | 325 | Durum Wheat | Spring wheat | 0.33 | 97.73 | SWHT |
| 39 | 326 | Corn | Spring wheat | 0.32 | 98.05 | CRSW |
| 40 | 330 | Millet | Fallow | 0.32 | 98.38 | HAY |
| 41 | 432 | Winter wheat | Sunflower | 0.30 | 98.68 | WWHT |
| 42 | 328 | Alfalfa | Corn | 0.28 | 98.96 | ALFA |
| 43 | 333 | Peas | Spring wheat | 0.27 | 99.23 | SYSW |
| 44 | 329 | Barley | Fallow | 0.26 | 99.49 | BARL |
| 45 | 331 | Spring wheat | Durum Wheat | 0.25 | 99.75 | SWHT |
| 46 | 332 | Barley | Spring wheat | 0.25 | 100.00 | BARL |

**Table S2.** Best estimates of hydrologic parameters at MORB

| Calibration Points | Definition | Allowable range | Culbertson | Sidney | Bismarck | Forestburg | Ashland | Sioux | Omaha | Desoto | Bagnell | Hermann |
| --- | --- | --- | --- | --- | --- | --- | --- | --- | --- | --- | --- | --- |
| CN2^a^ | Initial SCS runoff curve number for moisture condition II | -0.2-0.2 | 0.1 | -0.04 | 0.12 | -0.08 | -0.2 | -0.2 | 0.05 | -0.04 | -0.12 | 0.05 |
| ESCO | Soil evaporation compensation factor | 0-1 | 0.95 | 1 | 0.65 | 0.67 | 0.67 | 0.65 | 0.88 | 0.7 | 0.8 | 0.88 |
| EPCO | Plant uptake compensation factor | 0-1 | 0.9 | 0.7 | 0.8 | 0.8 | 0.6 | 0.4 | 0.4 | 0.5 | 0.1 | 0.4 |
| SOL_AWC(1)^a^ | Available water capacity of the soil layer (mm H_2_O /mm soil) | -0.2-0.2 | 0.2 | -0.16 | 0.05 | 0.13 | 0.17 | 0.12 | -0.09 | -0.14 | 0.2 | -0.09 |
| GW_DELAY | Groundwater delay time (days) | 0-500 | 150 | 230 | 130 | 150 | 280 | 150 | 190 | 100 | 250 | 190 |
| ALPHA_BF | Baseflow alpha factor (days) | 0-1 | 0.2 | 0.9 | 0.9 | 0.4 | 0.4 | 0.4 | 0.15 | 0.4 | 0.1 | 0.15 |
| GWQMN | Threshold depth of water in the shallow aquifer required for return flow to occur (mm H_2_O) | 0-5000 | 100 | 50 | 250 | 300 | 300 | 300 | 300 | 200 | 300 | 300 |
| GW_REVAP | Groundwater “revap” coefficient | 0.02-0.2 | 0.06 | 0.09 | 0.03 | 0.13 | 0.12 | 0.02 | 0.05 | 0.02 | 0.2 | 0.05 |
| RCHRG_DP | Deep aquifer percolation fraction | 0-1 | 0.45 | 0.5 | 0.4 | 0.4 | 0.4 | 0.45 | 0.4 | 0.5 | 0.4 | 0.4 |
| CH_K2 | Effective hydraulic conductivity in main channel | 0-500 | 15 | 55 | 15 | 20 | 5 | 25 | 30 | 25 | 5 | 30 |
| ALPHA_BNK | Baseflow alpha factor for bank storage | 0-1 | 0.09 | 0.1 | 0.12 | 0.04 | 0.18 | 0.12 | 0.25 | 0.1 | 0.05 | 0.25 |
| SFTMP | Snowfall temperature | -20-20 | 1 | | | | | | | | | |
| SMTMP | Snow melt base temperature | -20-20 | 2 | | | | | | | | | |
| SMFMX | Maximum melt rate for snow during year (occurs on summer solstice) | 0-20 | 4.5 | | | | | | | | | |
| SMFMN | Minimum melt rate for snow during the year (occurs on winter solstice) | 0-20 | 2.5 | | | | | | | | | |
| TIMP | Snow pack temperature lag factor | 0-1 | 0.7 | | | | | | | | | |

^a^ For CN2 and SOL_AWC(1), both allowable ranges and best estimates show the change from the default value as a fraction, for example, 0.1 corresponds to 10% increase and the allowable change of the parameters was within -0.2 and 0.2 of the default values.

**Table S3.** Best estimates of TSS, TN and TP parameters for MORB

|  | Calibration Points | Definition | Allowable range | Value |
| --- | --- | --- | --- | --- |
| TSS | USLE_P | USLE equation support pra | 0-1 | 0.65 |
|  | SPEXP | Exponent parameter for calculating sediment reentrained in channel sediment routing1 | 1-1.5 | 1.5 |
|  | SPCON | Linear parameter for calculating the maximum amount of sediment that can be reentrained during channel sediment routing | 0.0001-0.01 | 0.0012 |
|  | CH_COV1 | Channel erodibility factor | 0-0.6 | 0.3 |
|  | CH_COV2 | Channel cover factor | 0-1 | 0.5 |
| TN | ERORGN | Organic N enrichment ratio | 0-5 | 2.2 |
|  | NPERCO | Nitrogen percolation coefficient | 0-1 | 0.7 |
| TP | ERORGP | Organic P enrichment ratio | 0-5 | 1.6 |
|  | PPERCO | Phosphorus percolation coefficient | 10-17.5 | 15 |
